# Genetic Architecture of Group A Streptococcal Necrotizing Soft Tissue Infections in the Mouse

**DOI:** 10.1371/journal.ppat.1005732

**Published:** 2016-07-11

**Authors:** Karthickeyan Chella Krishnan, Santhosh Mukundan, Jeyashree Alagarsamy, Junguk Hur, Suba Nookala, Nikolai Siemens, Mattias Svensson, Ole Hyldegaard, Anna Norrby-Teglund, Malak Kotb

**Affiliations:** 1 Department of Molecular Genetics, Biochemistry and Microbiology, College of Medicine, University of Cincinnati, Cincinnati, Ohio, United States of America; 2 Department of Biomedical Sciences, School of Medicine and Health Sciences, University of North Dakota, Grand Forks, North Dakota, United States of America; 3 Karolinska Institutet, Centre for Infectious Medicine, Karolinska University Hospital, Stockholm, Sweden; 4 Department of Anaesthesia, Rigshospitalet, Copenhagen, Denmark; New York Medical College, UNITED STATES

## Abstract

Host genetic variations play an important role in several pathogenic diseases, and we have previously provided strong evidences that these genetic variations contribute significantly to differences in susceptibility and clinical outcomes of invasive Group A *Streptococcus* (GAS) infections, including sepsis and necrotizing soft tissue infections (NSTIs). Our initial studies with conventional mouse strains revealed that host genetic variations and sex differences play an important role in orchestrating the severity, susceptibility and outcomes of NSTIs. To understand the complex genetic architecture of NSTIs, we utilized an unbiased, forward systems genetics approach in an advanced recombinant inbred (ARI) panel of mouse strains (BXD). Through this approach, we uncovered interactions between host genetics, and other non-genetic cofactors including sex, age and body weight in determining susceptibility to NSTIs. We mapped three NSTIs-associated phenotypic traits (i.e., survival, percent weight change, and lesion size) to underlying host genetic variations by using the WebQTL tool, and identified four NSTIs-associated quantitative genetic loci (QTL) for survival on mouse chromosome (Chr) 2, for weight change on Chr 7, and for lesion size on Chr 6 and 18 respectively. These QTL harbor several polymorphic genes. Identification of multiple QTL highlighted the complexity of the host-pathogen interactions involved in NSTI pathogenesis. We then analyzed and rank-ordered host candidate genes in these QTL by using the QTLminer tool and then developed a list of 375 candidate genes on the basis of annotation data and biological relevance to NSTIs. Further differential expression analyses revealed 125 genes to be significantly differentially regulated in susceptible strains compared to their uninfected controls. Several of these genes are involved in innate immunity, inflammatory response, cell growth, development and proliferation, and apoptosis. Additional network analyses using ingenuity pathway analysis (IPA) of these 125 genes revealed interleukin-1 beta network as key network involved in modulating the differential susceptibility to GAS NSTIs.

## Author Summary

GAS bacteria are major human pathogens that are responsible for millions of infections worldwide, including severe and deadly NSTIs. Several studies have identified numerous GAS secreted virulence factors including proteases, DNases, and superantigens, which mediate several pathologic features of GAS NSTIs. However, the exact role of host genetic and/or nongenetic factors in GAS NSTIs has not been studied so far. To understand these contributions, we undertook the present study utilizing the ARI panel of BXD strains. We found that host genetic context and sex differences can modulate host-pathogen interplay and accordingly potentiate disease severity, manifestations, and outcomes. We also mapped the genetic susceptibility loci of GAS NSTIs to four mouse chromosomes, namely 2, 6, 7 and 18, harboring several polymorphic genes. We believe that these findings will be helpful in uncovering further regulatory events of host-mediated GAS pathogenesis that may occur once the pathogen becomes invasive.

## Introduction

The human skin acts as a barrier between the external environment and the body, protecting it from pathogens and regulating body temperature [1]. Nevertheless, skin infections occur. Group A *Streptococcus* (GAS) or *Streptococcus pyogenes* is one of the most common causative agents of human skin and severe soft tissue infections, which can range from uncomplicated impetigo and cellulitis to life-threatening necrotizing soft tissue infections (NSTIs) [2–9]. Our previous clinical investigations have shown that globally disseminated M1T1 GAS isolates can be isolated from patients with pharyngitis as well as from patients with severe invasive NSTIs, emphasizing the host factor contributions to the outcomes of invasive GAS infections [10]. Indeed, later studies revealed that allelic variations in human leukocyte antigen (HLA) class II haplotypes resulted in striking differences in severity and outcomes of invasive GAS infections *via* the differential presentations of GAS superantigens (SAgs) by class II HLAs to host T-cell receptor (TCR) Vβ elements; this finding further revealed a strong role for host factors in modulating GAS disease outcomes [11–14]. Nevertheless, in addition to SAgs, GAS harbors a wide array of virulence factors, including antiphagocytic M protein, fibronectin-binding proteins, proteases, DNases, and haemolysins, that can interact with a wide array of host factors besides HLA class II molecules during GAS pathogenesis. These unknown factors can influence susceptibility and severity of GAS NSTIs [15].

Although GAS causes NSTIs in humans, successful animal models have been developed through the use of conventional inbred and outbred mouse strains; however, mice are generally more resistant to GAS NSTIs than humans and thus require higher initial GAS inoculum than humans to develop NSTIs [16–23]. Our initial studies to understand the role of host genetic context in GAS NSTIs revealed that the DBA/2J (D2) mouse strain is more susceptible than is the C57BL/6J (B6) strain and that sex differences have a possible role in potentiating the severity of NSTIs [24]. However, the restricted genetic variations in these inbred strains led us to develop a better animal model for use in further in-depth investigations of the host genetic architecture for GAS NSTIs susceptibility. For this purpose and to identify additional host genetic and nongenetic factors, we undertook our present study utilizing an advanced recombinant inbred (ARI) mouse strains (BXD).

We applied a forward systems genetics approach to map quantitative trait loci (QTL) harboring genes that are most likely to be involved in modulating susceptibility/outcomes in a NSTIs model. A panel of distinct yet replenishable, genomically defined, and fully genotyped ARI mouse strains (BXD) [25], which were derived from B6 and D2 strains, were infected with an equal dose of M1T1 GAS bacteria [2, 10] and analyzed to detect variations in disease severity phenotypes. The quantitative phenotypes measured included survival, weight change kinetics, lesion size, bacterial load, and dissemination to organs.

In summary, we have established and optimized a murine model of NSTIs by utilizing a well-characterized and widely disseminated strain of GAS. Using this model, we mapped four NSTIs-associated QTL regions in the mouse and identified 125 polymorphic differentially expressed genes in representative susceptible BXD strains that have a high likelihood of modulating susceptibility to NSTIs. Finally, using network analyses, we narrow down interleukin-1 beta (*IL-1β)* as a key regulator in modulating NSTIs susceptibility.

## Results

### Differences in host- and sex-specific responses to GAS NSTIs

**Fig 1:**
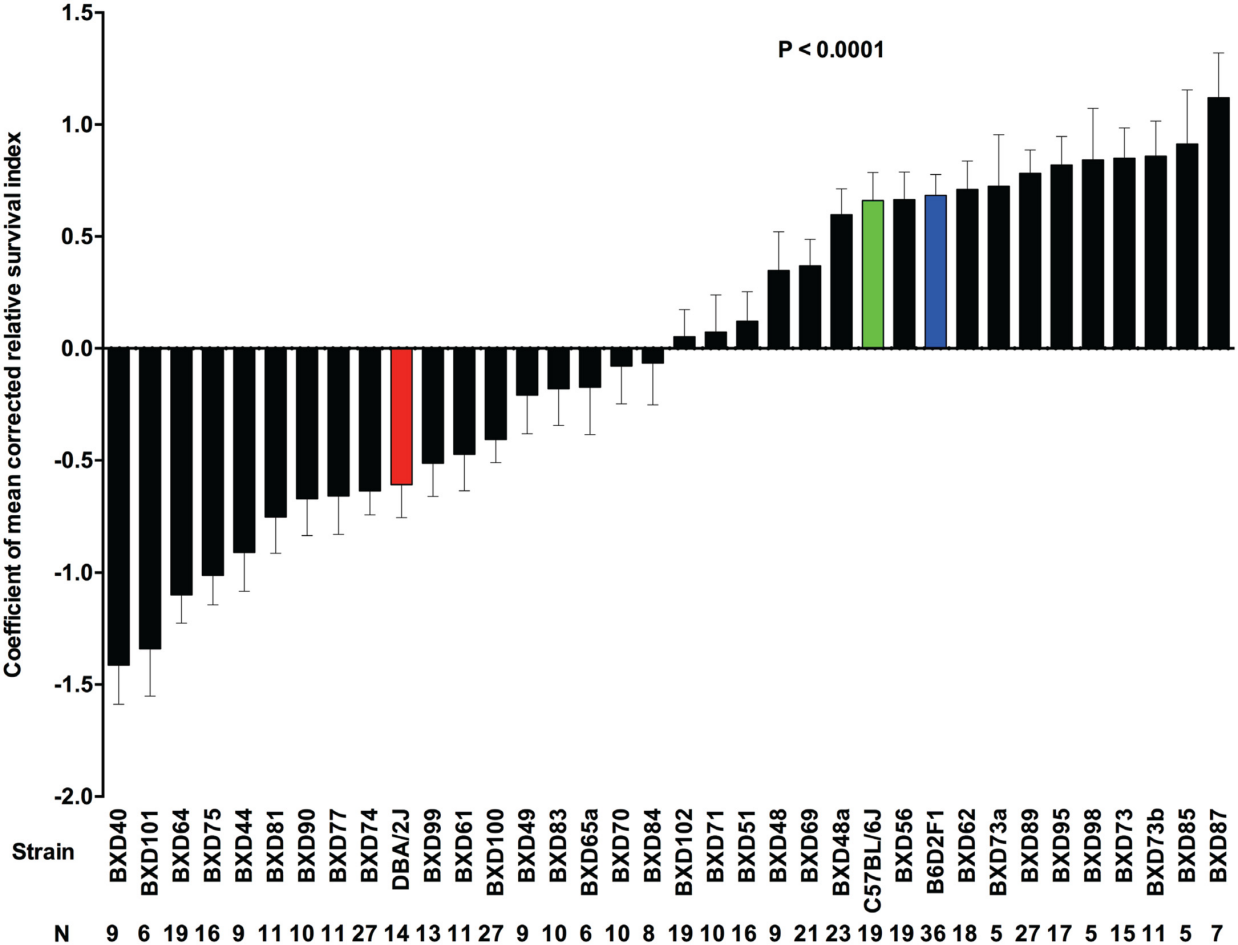
Differences in survival responses between BXD mice with GAS NSTIs. Survival within the 33 BXD (black bars), B6 (green bar), D2 (red bar) and their F1 (blue bar) strains is expressed as mean values of corrected relative survival indices. Data are rank-ordered with positive indices indicating increased survival and negative indices indicating decreased survival. Error bars indicate SEM. *P* values were calculated by GLM analysis using OLS ANOVA.

**Fig 2:**
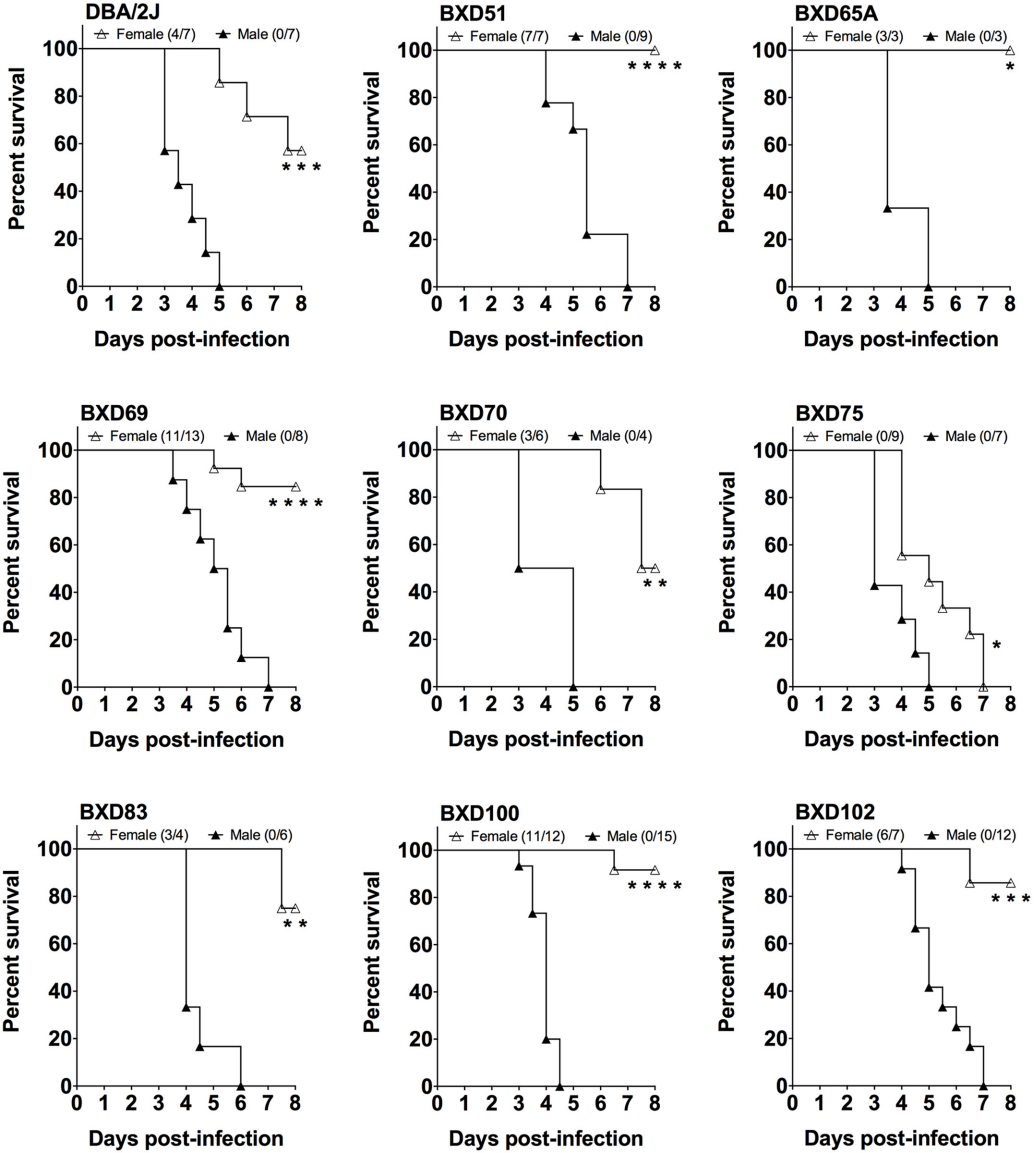
Sex-based differences in survival of D2 and BXD mice with GAS NSTIs. Survival curves for the male and female mice of D2 and other BXD strains, subcutaneously infected with an equal dose of GAS bacteria, are shown. Data presented are percent survival (*n* ≥ 3 for each sex). *P* values were calculated by log-rank (Mantel-Cox) tests. *P < 0.05, **P < 0.01, ***P < 0.001, ****P < 0.0001.

Our laboratory’s preliminary studies of subcutaneous GAS infections of conventional mouse strains determined that D2 mice are more susceptible to GAS NSTIs than are B6 mice; also D2 female mice are more resistant than are males of the same strain [24]. To further explore the relative contributions of host genetics, sex, and any other covariates to GAS NSTIs, we infected 33 BXD strains along with their ancestral parental strains (B6 and D2) and their F1 strains (B6D2F1) with an equal dose of GAS bacteria. Confirming our prior observations, the susceptibility of D2 mice was greater than that of B6 mice (Fig 1); also D2 female mice and female mice of other BXD strains were resistant in terms of survival (Fig 2). To further delineate the host genetic architecture of GAS NSTIs, we monitored three quantifiable phenotypic traits in mice of these infected strains: survival, weight change, and lesion size.

#### Trait 1: Survival

Survival data for each of the 33 BXD strains as well the B6, D2, and B6D2F1 strains were expressed as mean values of corrected relative survival indices (cRSI) as calculated in a general linear model (GLM) analysis. As expected, we observed significant differences (P ≤ 0.0001) in susceptibility to NSTIs across the BXD strains (Fig 1), some of which expressed phenotypes that were substantially different from those of their parental strains (B6 and D2). Thus, the results indicated the polygenic contributions to the observed phenotypic trait. In addition, the GLM analysis indicated that the covariates namely, mouse strain (*P* ≤ 0.0001), sex (*P* ≤ 0.0001), age (*P* ≤ 0.0001), and body weight (*P* = 0.0001) were significant predictors of survival (Table 1). Further, we estimated heritability (95.92%) to be the main effect attributable to survival (Table 2).

**Table 1:** GLM analysis of survival data.

Source	d.f.^a^	Sums of squares	Mean square	F-ratio	Probability
Constant	1	2383.78	2383.78	8575.60	≤ 0.0001
Strain	35	228.51	6.53^b^	23.49	≤ 0.0001
Sex	1	18.75	18.75	67.46	≤ 0.0001
Age	1	12.26	12.26	44.11	≤ 0.0001
Body weight	1	4.07	4.07	14.65	0.0001
Error	469	130.37	0.28^b^		
Total	507	441.88			

^a^ d.f.—number of degrees of freedom.

^b^ Used for broad sense heritability calculation.

**Table 2:** Broad sense heritability of NSTIs phenotypic traits.

Trait	Trait ID	Heritability^a^
Survival	17524	95.92%
Day 1 percent WT change	17523	76.43%
Day 2 percent WT change	17522	79.25%
Day 3 percent WT change	17521	85.52%
Day 4 percent WT change	17520	85.81%
Lesion size	17525	80.98%

^a^ Calculated from the respective GLM ANOVA table

#### Trait 2: Weight change kinetics

**Fig 3:**
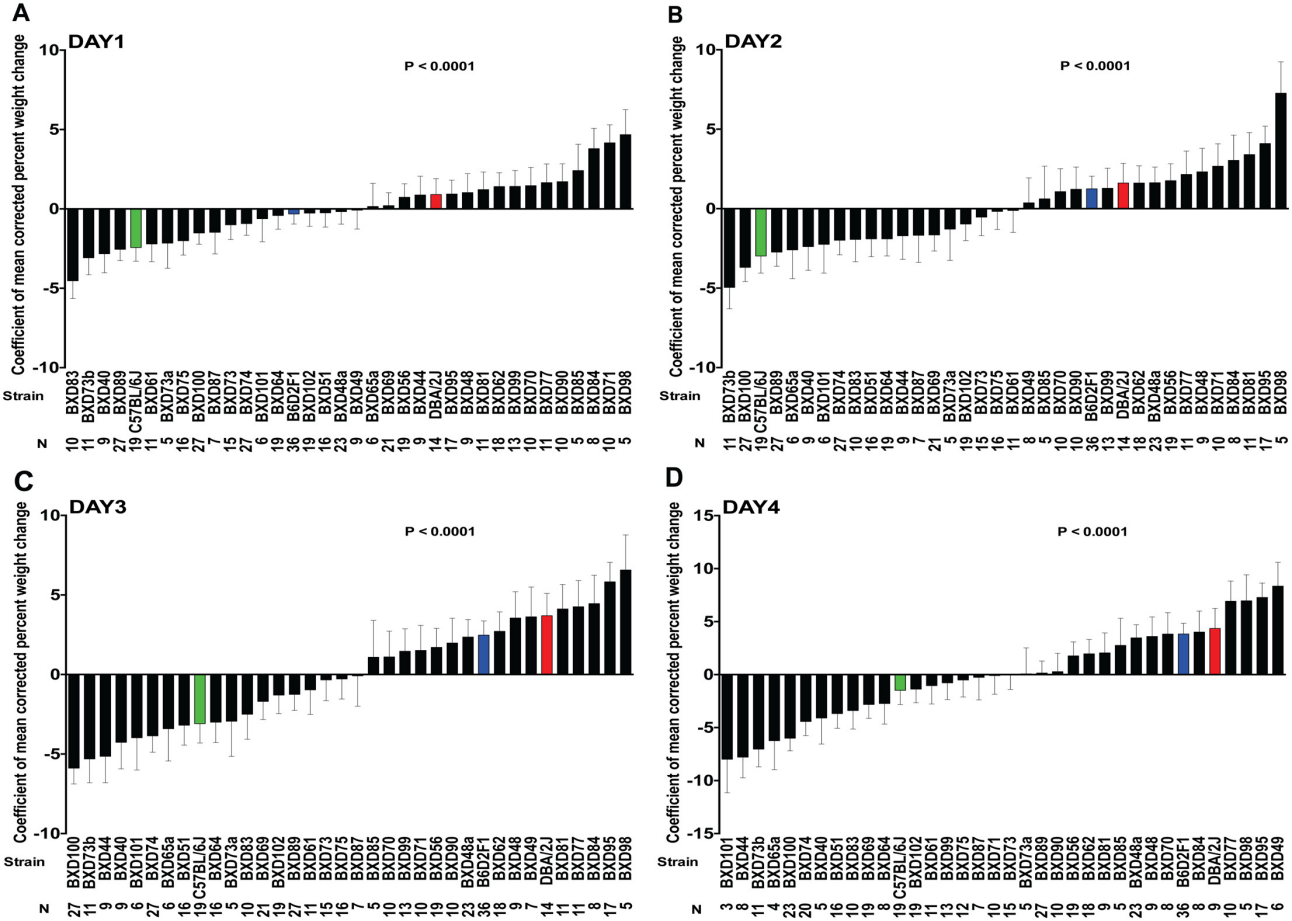
Differences in weight change between BXD mice during the first four days of GAS NSTIs. Percent weight changes on (A) Day 1, (B) Day 2, (C) Day 3, and (D) Day 4 percent weight change results across 33 BXD (black bars), B6 (green bar), D2 (red bar) and their F1 (blue bar) strains are expressed as mean values of corrected percent weight change. Data are rank-ordered with negative values indicating maximum weight loss and positive values indicating minimal weight loss. Error bars indicate SEM. *P* values were calculated by GLM analysis using OLS ANOVA.

Using a similar GLM analysis, we calculated the mean values of corrected percent weight change for the first four days after infection. We observed significant differences among the different BXD strains and their ancestral parents (Fig 3). Further, GLM analysis revealed that mouse strain (*P* ≤ 0.0001) and body weight (*P* ≤ 0.0001) were significant predictors of percent weight change in all four days (Table 3). Heritability (76.43%– 85.81%) was estimated to be the main effect on percent weight change, and the effect increased from day 1 through day 4 (Table 2).

**Table 3:** GLM analysis of percent weight change kinetics data.

Source	d.f.^a^	Sums of squares	Mean square	F-ratio	Probability
(A) Day 1 percent weight change					
Constant	1	15831.50	15831.50	1216.80	≤ 0.0001
Strain	35	1476.53	42.19^b^	3.24	≤ 0.0001
Sex	1	112.37	112.37	8.64	0.0035
Age	1	188.98	188.98	14.53	0.0002
Body weight	1	143.05	143.05	11.00	0.0010
Error	469	6101.88	13.01^b^		
Total	507	8034.98			
(B) Day 2 percent weight change					
Constant	1	39813.80	39813.80	1966.10	≤ 0.0001
Strain	35	2706.42	77.33^b^	3.82	≤ 0.0001
Sex	1	47.67	47.67	2.35	0.1257
Age	1	138.20	138.20	6.82	0.0093
Body weight	1	449.09	449.09	22.18	≤ 0.0001
Error	468	9477.21	20.25^b^		
Total	506	12802.20			
(C) Day 3 percent weight change					
Constant	1	46661.70	46661.70	1837.80	≤ 0.0001
Strain	35	5247.23	149.92^b^	5.90	≤ 0.0001
Sex	1	13.17	13.17	0.52	0.4717
Age	1	66.78	66.78	2.63	0.1055
Body weight	1	888.40	888.40	34.99	≤ 0.0001
Error	464	11780.90	25.39^b^		
Total	502	19492.00			
(D) Day 4 percent weight change					
Constant	1	54837.50	54837.50	1764.50	≤ 0.0001
Strain	35	6576.75	187.91^b^	6.05	≤ 0.0001
Sex	1	6.90	6.90	0.22	0.6377
Age	1	84.05	84.05	2.70	0.1008
Body weight	1	1671.64	1671.64	53.79	≤ 0.0001
Error	418	12990.90	31.08^b^		
Total	456	23260.50			

GLM analysis of percent weight change data for (A) day 1, (B) day 2, (C) day 3, and (D) day 4 post-infection in a GAS NSTIs model.

^a^ d.f.—number of degrees of freedom.

^b^ Used for broad sense heritability calculation.

#### Trait 3: Lesion size

**Fig 4:**
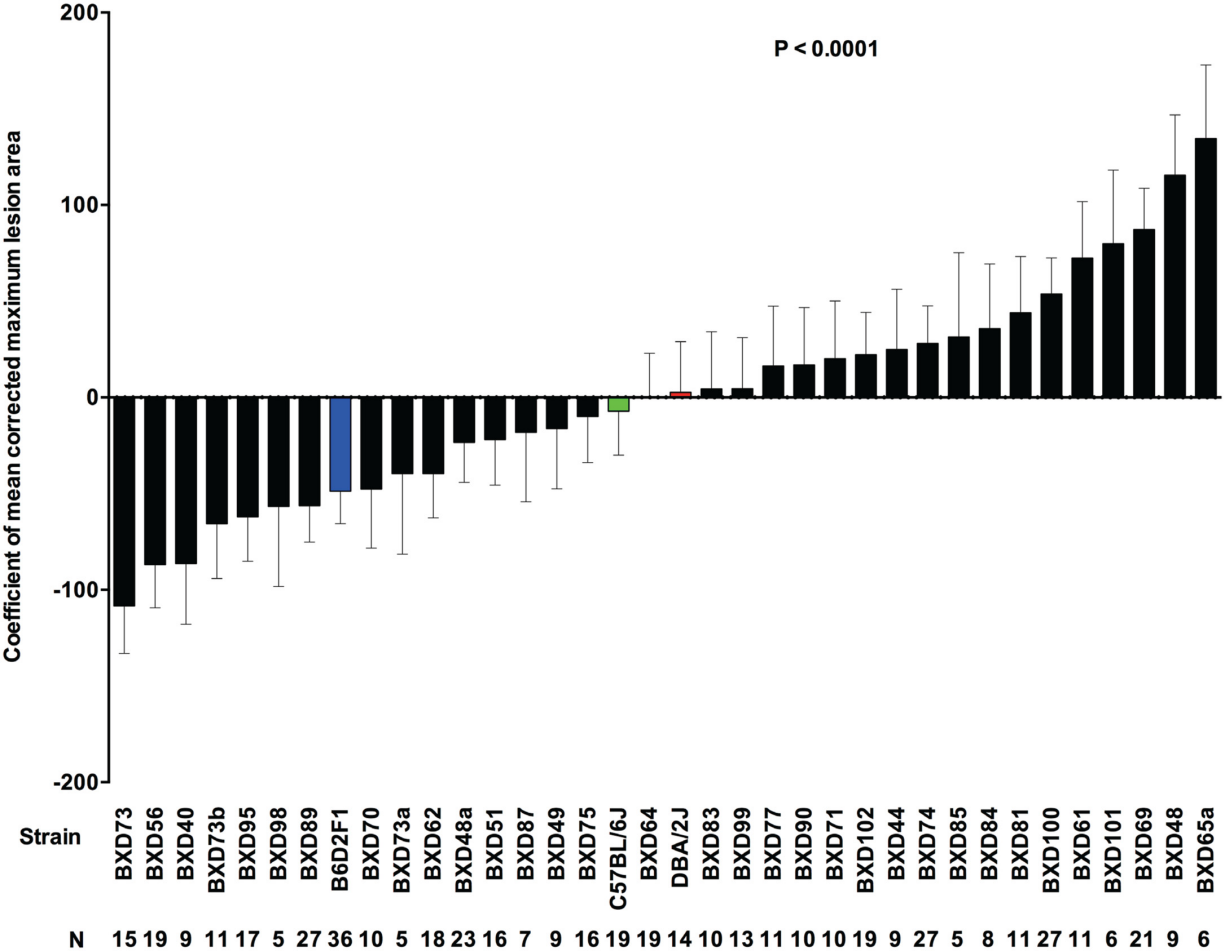
Differences in lesion size among BXD mice with GAS NSTIs. Lesion sizes in 33 BXD (black bars), B6 (green bar), D2 (red bar) and their F1 (blue bar) strains are expressed as mean values of corrected maximum lesion area. Data are rank-ordered with positive values indicating increased lesion sizes and negative values indicating reduced lesion size. Error bars indicate SEM. *P* values were calculated by GLM analysis using OLS ANOVA.

Mean values of corrected maximum lesion area were calculated in the GLM analysis, and there was a significant difference between the strains tested (Fig 4). Mouse strain (*P* ≤ 0.0001) and body weight (*P* = 0.0176) were major predictors of lesion size (Table 4), along with heritability (80.98%), which was estimated as the main effect on lesion size (Table 2).

**Table 4:** GLM analysis of lesion size data.

Source	d.f.^a^	Sums of squares	Mean square	F-ratio	Probability
Constant	1	12341793.00	12341793.00	1358.40	≤ 0.0001
Strain	35	1353828.00	38680.80^b^	4.26	≤ 0.0001
Sex	1	18341.50	18341.50	2.02	0.1560
Age	1	22.86	22.86	0.00	0.9600
Body weight	1	51587.50	51587.50	5.68	0.0176
Error	469	4261180.00	9085.67^b^		
Total	507	5889918.00			

^a^ d.f.—number of degrees of freedom.

^b^ Used for broad sense heritability calculation.

### Genome-wide linkage scans mapping host genetic loci modulating quantitative phenotypic NSTI traits

To identify significant QTL that modulate the observed phenotypes, we performed genome-wide linkage scans (GWLS) by mapping quantitative phenotypic traits to the BXD mouse genotypes available from the Gene Network (GN).

#### Trait 1: Survival

**Fig 5:**
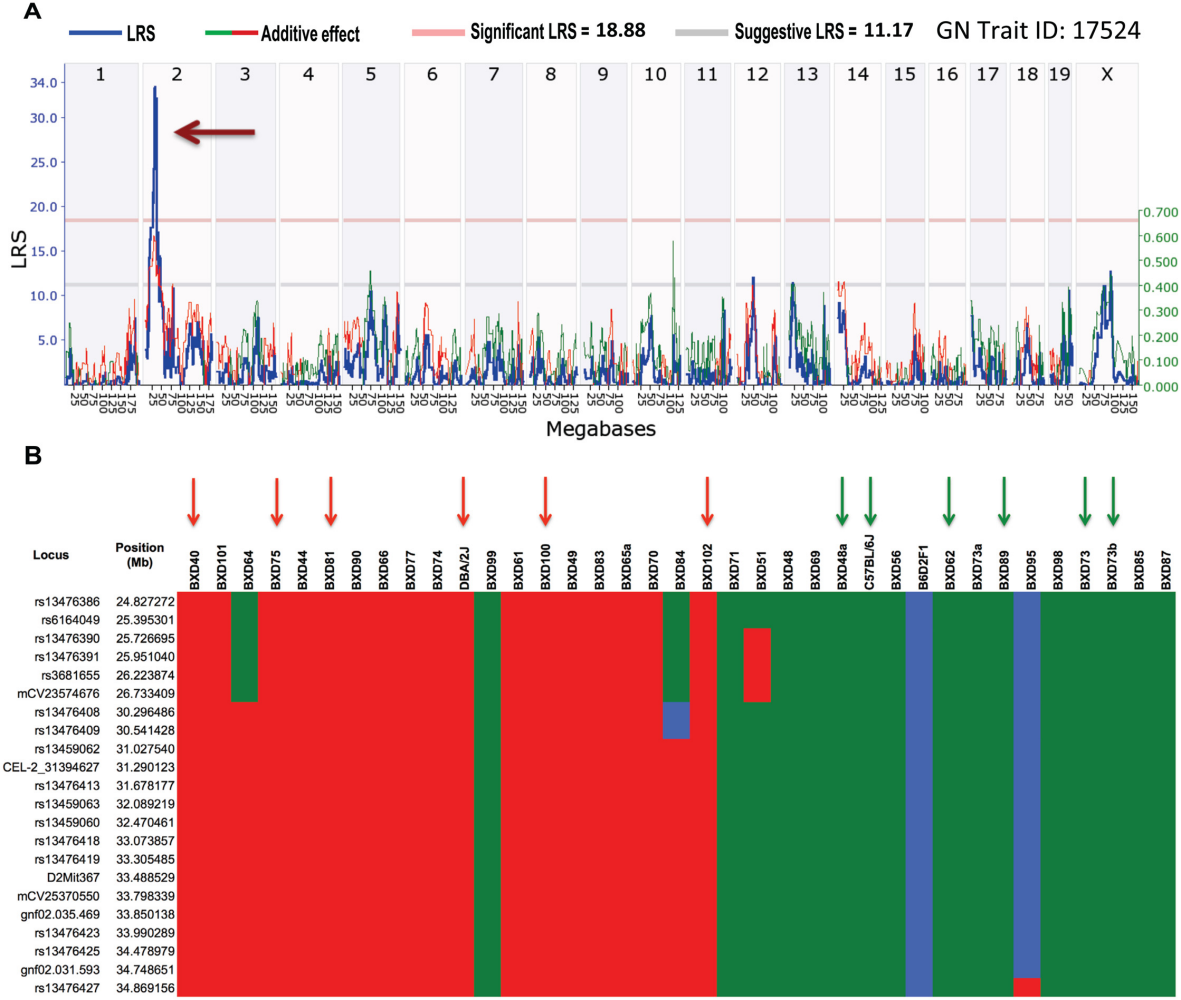
QTL mapping and haplotype analysis for survival against GAS NSTIs. (A) Genome-wide interval mapping of survival data (expressed as cRSI across BXD and parental strains) reveal a significant QTL on mouse Chr 2 (brown arrow). Red and gray horizontal lines indicate significant and suggestive LRS thresholds, respectively. (B) Haplotype analysis of the QTL region between 24.5 and 35 Mb on mouse Chr 2 is shown. BXD strains were rank-ordered on the basis of their cRSI values from susceptible to more resistant. Red and green bars (within each loci/position) indicate D and B alleles, respectively, whereas blue bars indicate heterozygous alleles. BXD cohorts harboring either D (red arrows) or B (green arrows) haplotypes within the QTL region and their parental strains selected for *in silico* validation analyses are indicated.

Our survival data (GN Trait ID: 17524) mapped to a single highly significant QTL between 24.5 and 35 Mb on mouse Chr 2; the peak likelihood ratio statistic (LRS) was 33.6 (Fig 5A). Ensuing haplotype analysis revealed that D haplotypes contributed to increased mortality (Fig 5B).

#### Trait 2: Weight change kinetics

**Fig 6:**
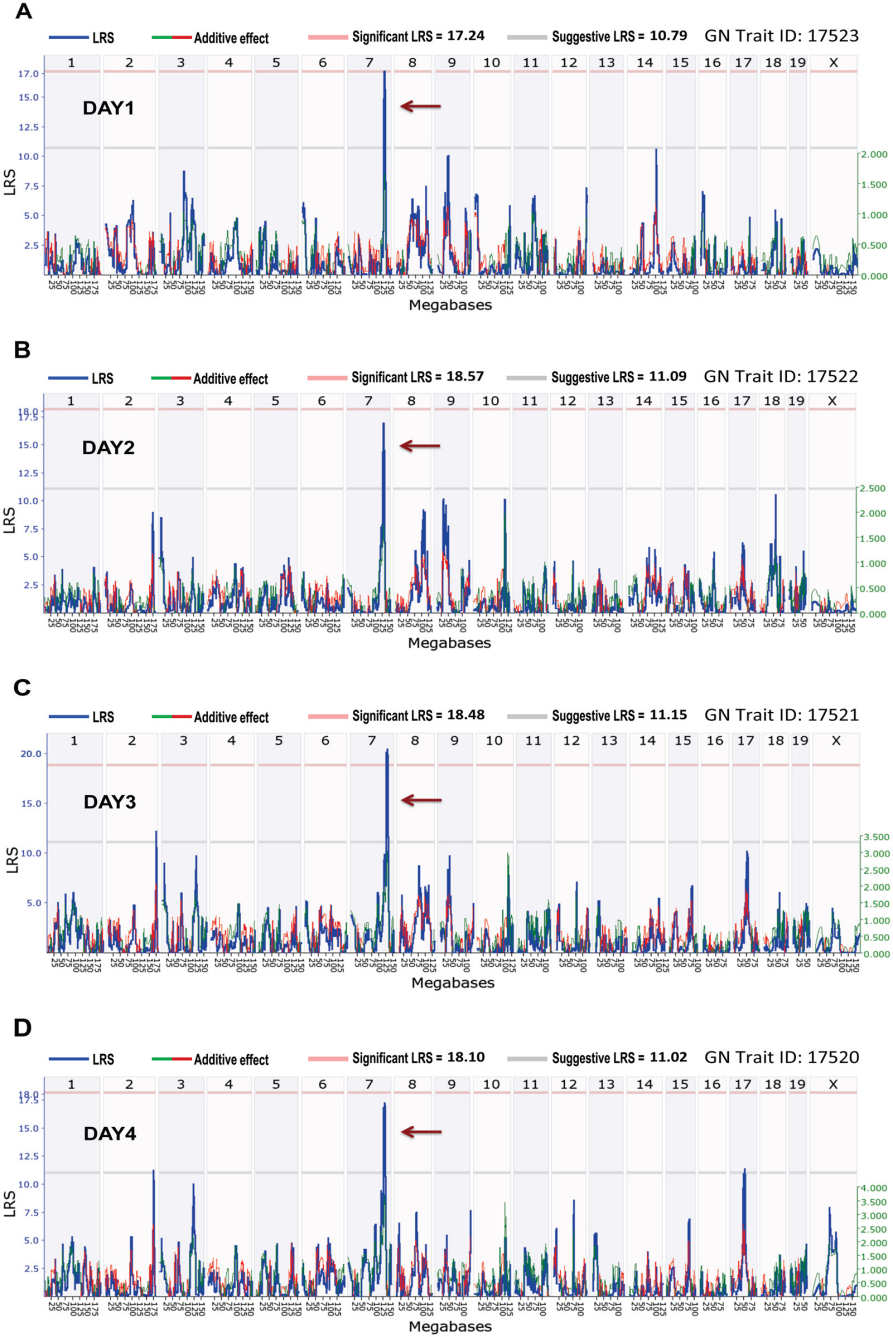
QTL mapping for weight change kinetics during the first four days of GAS NSTIs. Genome-wide interval mapping for percent weight change on (A) Day 1, (B) Day 2, (C) Day 3, and (4) Day 4 revealed a consistent QTL on mouse Chr 7 (brown arrow). Red and gray horizontal lines indicate significant and suggestive LRS thresholds, respectively.

**Fig 7:**
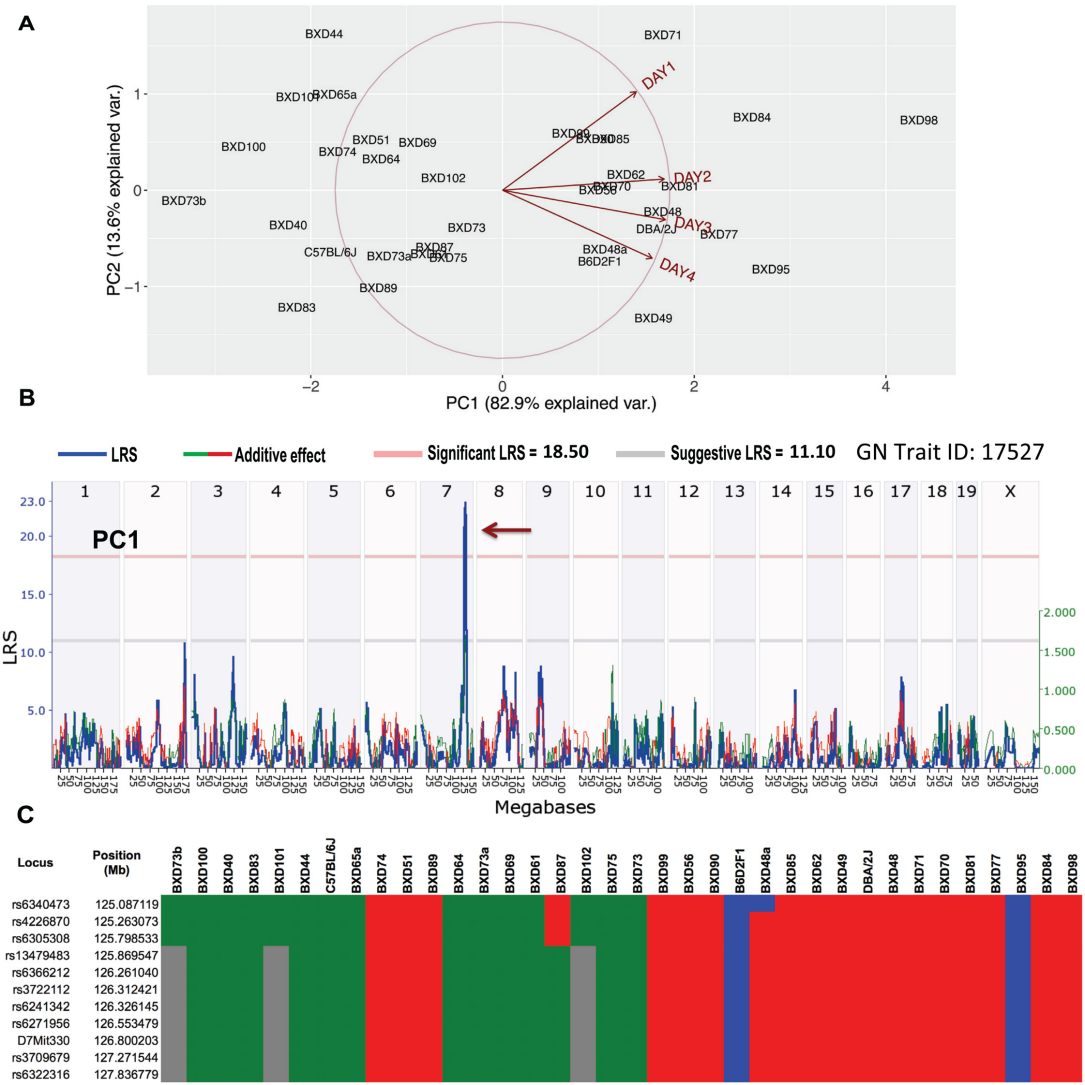
Principal component analysis, QTL mapping, and haplotype analysis for weight change kinetics during the first four days of GAS NSTIs. (A) PCA ggbiplot display the first two principal components (PC1 and PC2) of four non-independent percent weight change measurements. As shown, PC1 explains most of the variances between the four data. (B) Genome-wide interval mapping for PC1 revealed a significant QTL on mouse Chr 7 (brown arrow). Red and gray horizontal lines indicate significant and suggestive LRS thresholds, respectively. (C) Results of the haplotype analysis of the QTL region between 125 and 131 Mb on mouse Chr 7 are shown. BXD strains were rank-ordered on the basis of their PC1 values from maximum weight loss to minimum. Red and green bars (within each loci/position) indicate D and B alleles, respectively, whereas blue and gray bars indicate heterozygous and unknown alleles, respectively.

While mapping the percent weight change data for days 1–4 (GN Trait ID: 17520–17523), we observed several volatile non-significant QTL along with a single consistent QTL on mouse Chr 7 (Fig 6). This finding is not uncommon as other investigations of weight loss in BXD mice with H1N1 influenza [26] or *Burkholderia pseudomallei* [27] infections have revealed multiple QTL initially that were reduced when subjected to principal component analysis (PCA). Therefore, to reduce the noise from these four non-independent measurements, PCA was performed. One major component—PC1, which covers 82.9% of the variances, was identified (Fig 7A). GWLS mapped PC1 (GN Trait ID: 17527) to a single highly significant QTL that had a peak LRS of 22.5 and was located between 125 and 131 Mb on mouse Chr 7 (Fig 7B). Further haplotype analysis revealed the contribution of B haplotypes to the increase in percent weight loss (Fig 7C).

#### Trait 3: Lesion size

**Fig 8:**
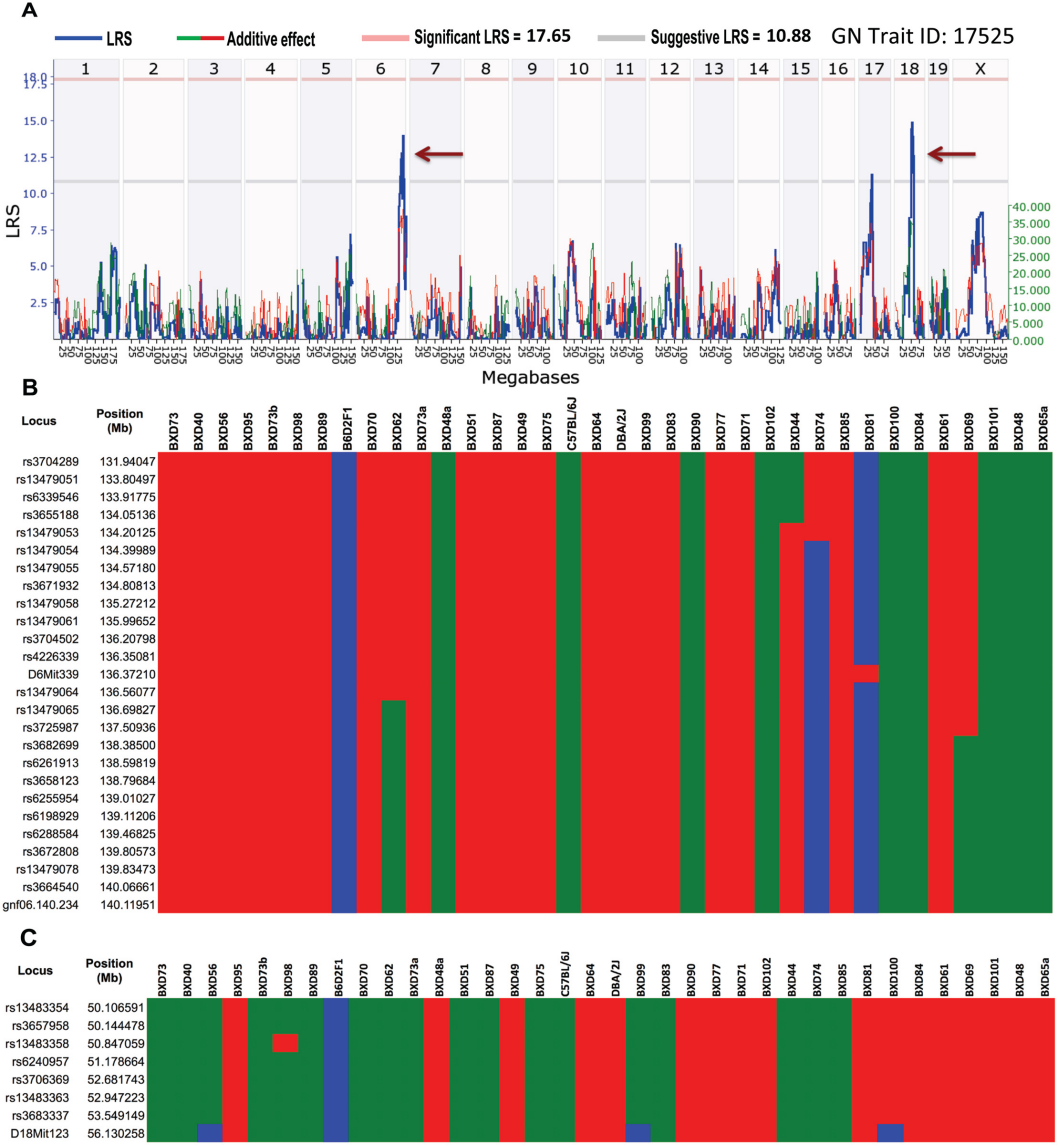
QTL mapping and haplotype analysis for maximum lesion size associated with GAS NSTIs. (A) Genome-wide interval mapping for lesion size revealed two suggestive QTLs on mouse Chr 6 and 18 (brown arrows). Red and gray horizontal lines indicate significant and suggestive LRS thresholds, respectively. Results of the haplotype analysis of the QTL region (B) between 131.6 and 141.8 Mb on mouse Chr 6 and (C) between 49.5 and 56.3 Mb on Chr 18 are shown. BXD strains are rank-ordered on the basis of lesion sizes (from larger to smaller lesions). Red and green bars (within each loci/position) indicate D and B alleles, respectively, and blue bars indicate heterozygous alleles.

Using GWLS, we mapped our lesion data (GN Trait ID: 17525) to two suggestive QTL between 131.6 and 141.8 Mb on Chr 6 and between 49.5 and 56.3 Mb on Chr 18; the peak LRS was 14.9 (Fig 8A). Haplotype analyses of Chr 6 revealed B haplotypes (Fig 8B), whereas analyses of Chr 18 revealed D haplotypes that contributed to the increase in lesion size (Fig 8C).

### *In silico* prediction and validation of BXD susceptibility toward GAS NSTIs

**Fig 9:**
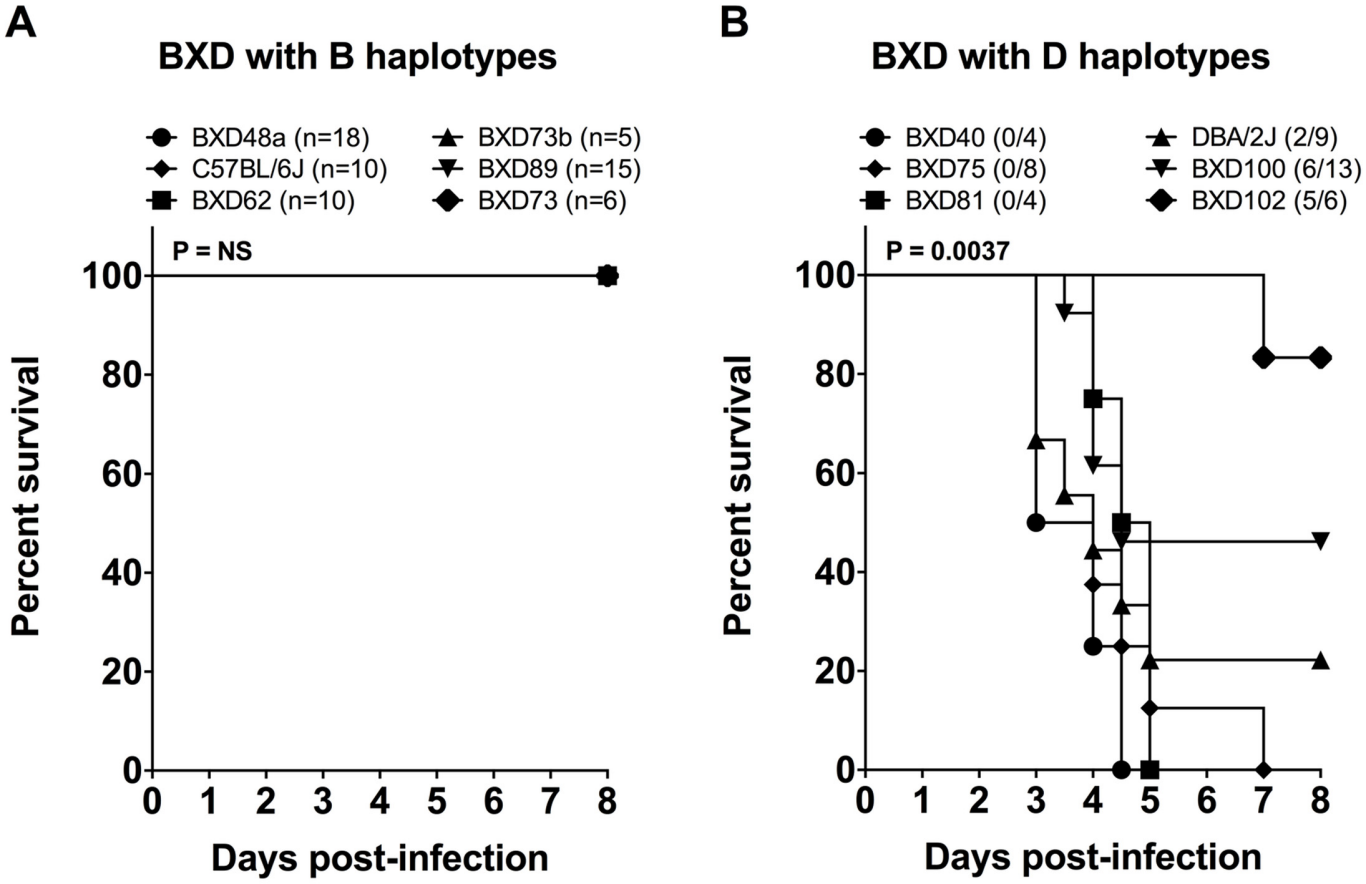
Differences in survival between BXD mice with NSTIs harboring either B or D haplotypes within their survival QTL regions. Survival curves for the selected BXD cohorts harboring either (A) B haplotypes or (B) D haplotypes within their survival QTL regions on mouse Chr 2 and survival curves for their parental strains are shown. Results are percent survival (*n* ≥ 4 for each group). *P* values were calculated by log-rank (Mantel-Cox) tests.

To validate our mapped survival QTL, we selectively infected a BXD strain with a D (or B) haplotype within the observed QTL region and determined whether such mice exhibited a susceptible phenotype such as increased mortality associated with increased bacterial burden or a resistant phenotype such as reduced mortality associated with reduced bacterial burden. Based on the mapping of the survival phenotype to the QTL (Fig 5B), we chose 10 BXD strains with either a B or a D haplotype between 24.5 and 35 Mb at Chr 2 and infected them and their ancestral parents (B6 and D2) with an equal dose of GAS bacteria. All the BXD strains with B haplotypes within the survival QTL (and the B6 strain) were resistant in terms of survival (Fig 9A), whereas the BXD strains with D haplotypes (and the D2 strain) were found to be differentially susceptible in terms of survival (Fig 9B). These differences in survival confirmed the role of other confounding nongenetic cofactors (including sex, age, and body weight).

**Fig 10:**
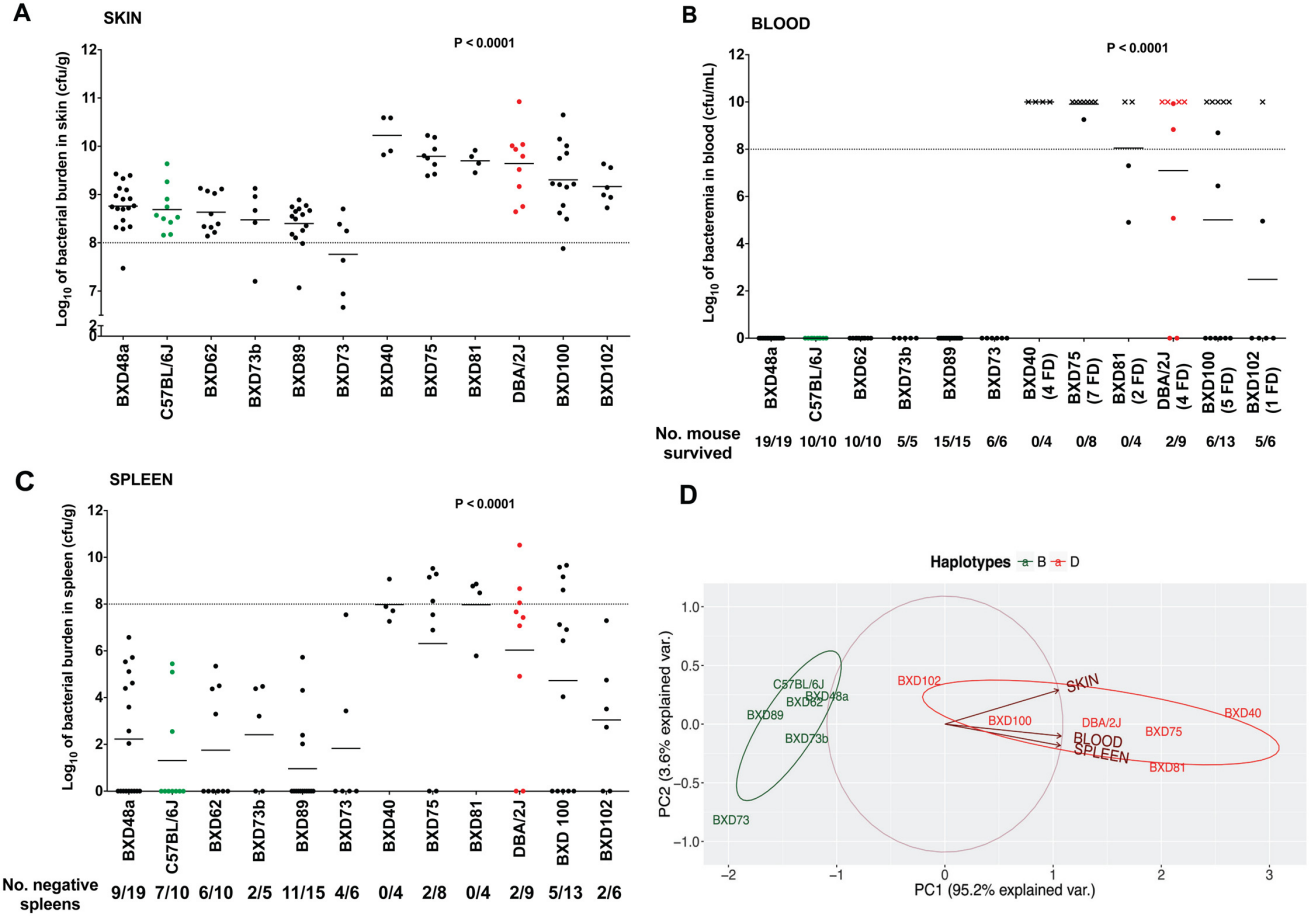
Differences in bacterial loads and dissemination between BXD mice harboring either B or D haplotypes within their survival QTL regions. Shown are the bacterial loads in (A) skin, (B) blood, and (C) spleen collected during the seven-day infection timeline, from surviving and dead mice of BXD strains harboring either a B (left) or D (right) haplotypes within their survival QTL regions. Similar data from their parental strains (B6 –green and D2 –red) are also shown. BXD mice are rank-ordered, within their groups, based on their bacterial counts. Each mouse is represented by a symbol with the horizontal bar representing the mean, and the horizontal dotted line indicating the inoculum given. Bacteremia counts for dead mice (cross symbols) for which blood collections were missed were assigned an arbitrary value of 10^10^ CFU/mL (a value near the maximum bacteremia count). Data presented are log-transformed bacterial loads (*n* ≥ 4 for each strain). *P* values were calculated by one-way ANOVA. (D) The PCA ggbiplot displays the first two principal components (PC1 and PC2) of three non-independent bacterial measurements. BXD strains harboring D and B haplotypes are indicated by red and green, respectively. The BXD strains are grouped together on the basis of their haplotypes.

Next, we looked for any marked differences in the bacterial load in skin and blood and in bacterial dissemination between the BXD mice with either the B or the D haplotypes within the survival QTL. All BXD strains with D haplotypes and the D2 strain contained significantly greater bacterial loads in skin, greater bacteremia, and greater dissemination to the spleen than did the BXD strains with B haplotypes and the B6 strain (Fig 10A–10C). PCA of these three non-independent bacterial load measurements revealed one major component, PC1, which covered 95.2% of the variances (Fig 10D). The BXD strains were grouped together on the basis of their haplotypes, with the BXD strains with a D haplotype representing a positive group in terms of increased bacterial load and dissemination.

### Identification of differentially expressed host candidate genes and gene networks within the mapped loci that contribute to GAS NSTI susceptibility

GWLS of three monitored phenotypic traits relevant to NSTIs (survival, percent weight change, and lesion size) revealed four QTL that contain 516 genes on mouse Chr 2 (276 genes), 7 (98 genes), 6 (106 genes), and 18 (36 genes). From this extensive gene list, we wanted to further narrow down and identify potential host candidate genes. First, we used the QTLminer tool available in GN [28] to investigate our QTL and rank-order the genes (rank 0–4) based on (i) gene annotation data (including gene name, description, Gene Ontology terms), (ii) normal gene expression datasets (adipose, muscle, and spleen mRNA), (iii) gene polymorphism data (non-synonymous single nucleotide polymorphisms [nsSNPs] found between the ancestral parental B6 and D2 strains and insertion or deletion of bases [INDELs] found in the BXD strains), and (iv) genes that have local genetic control (cis-eQTLs). From these rank-ordered genes, we shortlisted 375 host candidate genes on mouse Chr 2 (224 genes), 7 (74 genes), 6 (55 genes), and 18 (22 genes) on the basis of their annotation data and biological relevance to GAS NSTIs (S1–S4 Tables).

Next, these 375 shortlisted genes were further evaluated for their differential expression profiles at 48h post-infection, between the representative susceptible (BXD 40 and 64) and resistant (BXD 73 and 87) BXD strains compared with their respective uninfected controls, using custom designed 384-well real time PCR plates with PrimePCR SYBR Green assays as described in the methods section. Accordingly, we found that 125 genes were significantly (FDR < 0.10) differentially expressed (±1.5 fold regulation as threshold) in susceptible strains. However, none of the tested genes were significantly differentially regulated in resistant strains compared to their uninfected controls. Detailed lists of all these genes along with their expression fold regulation values are given in S5–S7 Tables.

**Fig 11:**
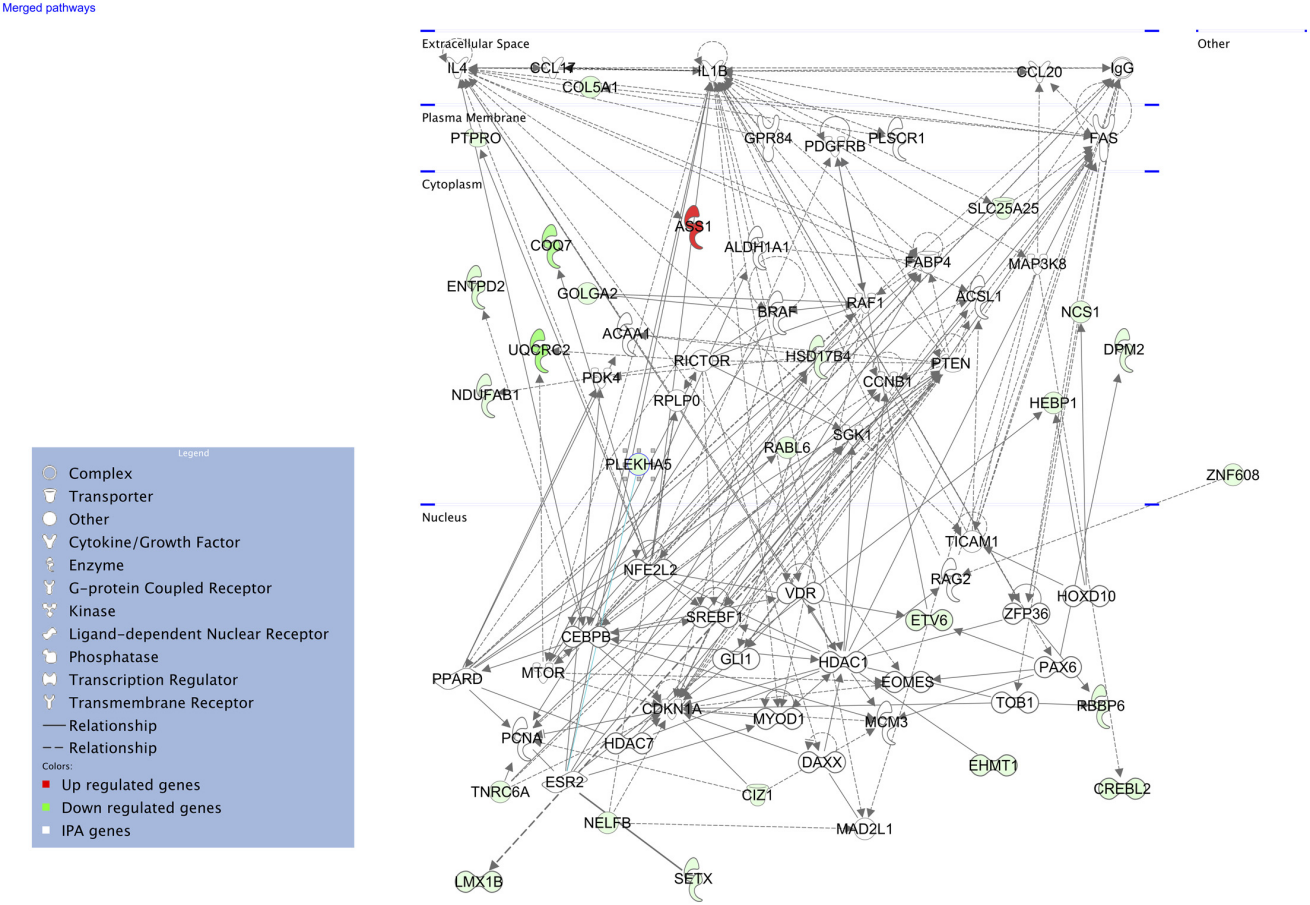
Functional gene network modulating GAS NSTIs. Graphical representation of molecular interactions between significantly up regulated (red) or down regulated (green) genes within the QTL in the susceptible BXD strains, highlighting the central role of interleukin-1 beta (*IL-1β*) as a key regulator in modulating GAS NSTIs. Genes are represented as nodes, and the biological relationship between two nodes is represented as line, solid lines represent direct interactions and dashed lines represent indirect interactions. All the gene names are listed in S8 Table. The networks were generated through the use of QIAGEN’s Ingenuity Pathway Analysis (IPA, QIAGEN Redwood City, http://www.qiagen.com/ingenuity).

**Fig 12:**
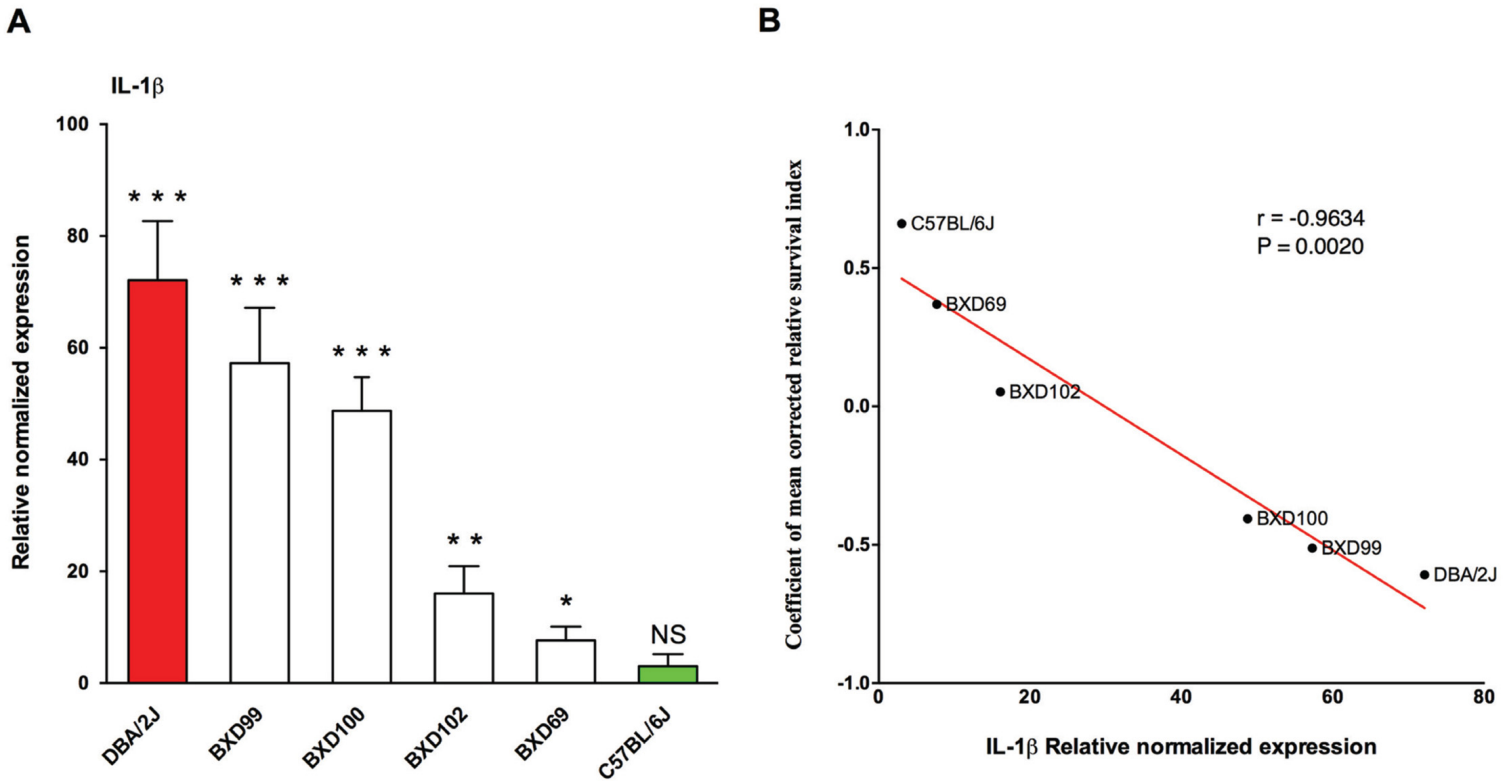
Expression of *IL-1β* in different BXD strains. (A) Shown are the relative normalized *IL-1β* mRNA expression at the infected site of parental strains (B6 –green and D2 –red) as well as representative BXD strains (BXD 69, 99, 100 and 102), selected based on their cRSI (survival index), compared to their respective uninfected controls. The data represent mean values ± SD (*n* = 3 for each strain). *P* values were calculated through student’s t-test. *P < 0.05, **P < 0.01, ***P < 0.001, NS—non significant. (B) Correlation of cRSI (survival index) with *IL-1β* mRNA expression. Regression line is shown in red. r, represents pearson correlation coefficient; P, represents *P* value.

Finally, we parsed these significantly (FDR < 0.10) differentially expressed (±1.5 fold regulation as threshold) 125 genes into pathways using Ingenuity Pathway Analysis (IPA). Through this, we extracted two high-scoring networks, which are shown merged in Fig 11, comprising genes that are related to cancer, cell death and survival, inflammatory response, organismal injury and abnormalities, and tissue morphology. Based on these networks, interleukin-1 β (*IL-1β*) was likely to be the key response molecule regulating susceptibility to GAS NSTIs. We then tested its differential regulation at 48h post-infection, in both ancestral strains (B6 and D2) and between the representative BXD strains (BXD 69, 99, 100 and 102) compared with their respective uninfected controls. These BXD strains were selected based on their respective cRSI (survival index) as shown in Fig 1 and covered a wide range of survival indices between the two ancestral strains. As expected, we observed that *IL-1β* was significantly up regulated in D2 parental strains compared to B6 (Fig 12A). In addition, we observed that the *IL-1β* expression was significantly correlated with infection severity as shown in Fig 12B, wherein we found that strains with lower cRSI values (decreased survival) had significantly higher *IL-1β* expression compared to their respective uninfected controls and *vive versa*.

### Validation of interleukin-1 beta (*IL-1β*) as a potential key regulator in GAS NSTI pathogenesis

**Fig 13:**
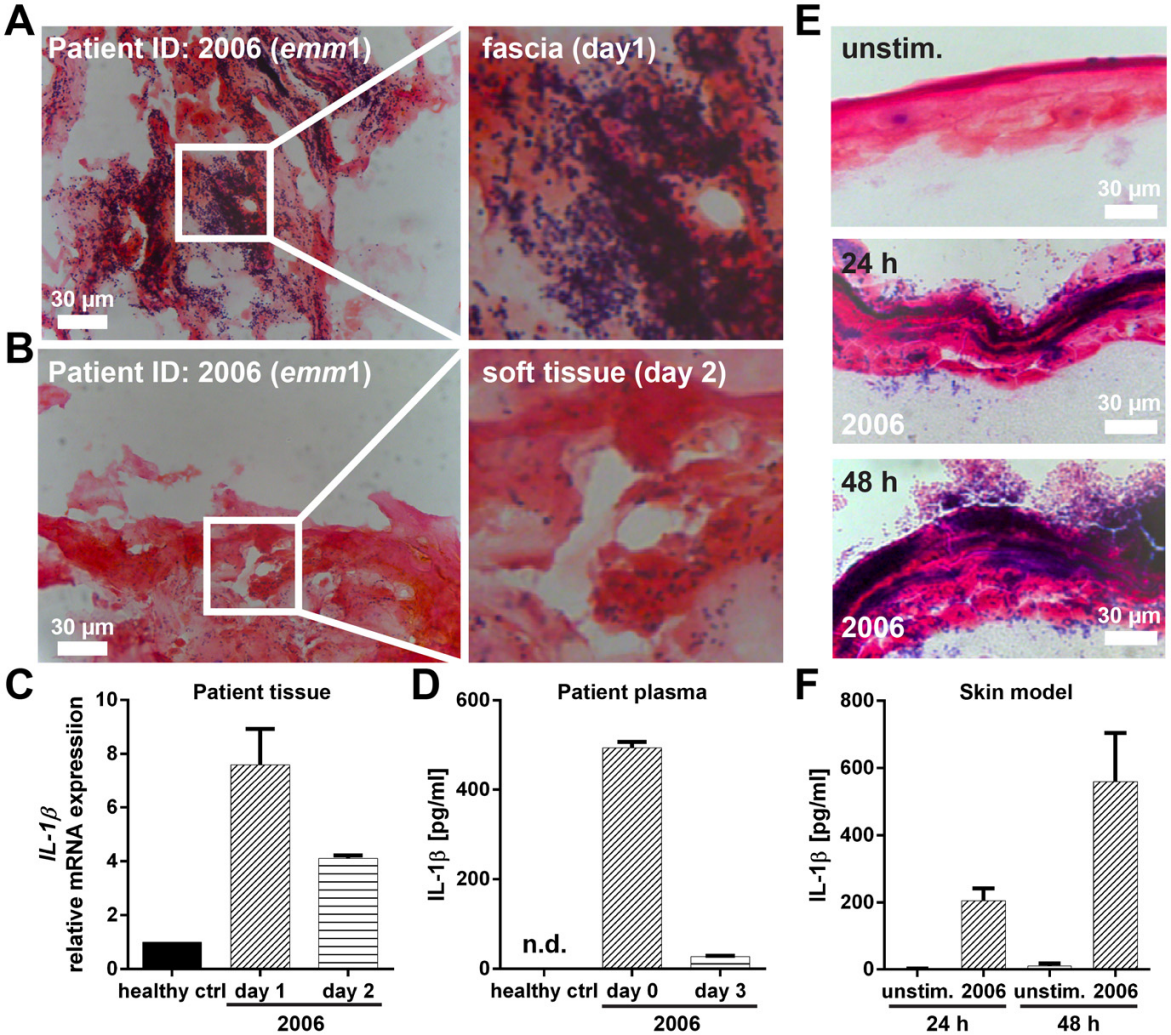
Expression of IL-1β in patient tissue and skin tissue models. Identification of bacteria in patient biopsies was visualized by conventional Gram staining. Representative images of tissue sections from patient 2006 at (A) day 1 and (B) day 2 are shown. (C) Relative *IL-1β* mRNA expression at the local site of infection. Mean values ± SD from biopsies of two healthy volunteers and two technical replica of the patient biopsy at indicated days are shown. (D) IL-1β in plasma samples collected from patient 2006 and three healthy volunteers are shown. Mean values ± SD from two technical replica of patient 2006 on indicated days are shown. (E) Representative images of Gram stained skin tissue models 24 and 48 hours after infection. (F) Detection of IL-1β levels in skin model culture supernatants at indicated time points. The data represent mean values ± SD from three independent experiments (n = 3).

To further validate *IL-1β* as a potential key factor involved in susceptibility to GAS NSTI, we conducted a proof-of-concept experiment in which plasma samples and tissue biopsies from an M1 GAS infected NSTI patient (2006), as well as samples from an *in vitro* experimental model of human skin infected with the patient’s isolate were analyzed. Light microscopy analysis of Gram-stained tissue biopsies collected on days 1 and 2 identified Gram-positive cocci in the affected tissue with a massive bacterial load noted in the day 1 biopsy while substantially fewer cocci were detected in the day 2 biopsies (Fig 13A and 13B). Analyses of *IL-1β* gene expression showed an increased expression in patient biopsies as compared to biopsies from healthy controls (Fig 13C). In line with the bacterial load, *IL-1β* gene expression decreased in the day 2 biopsy. Similarly, analyses of systemic IL-1β revealed high levels in patient plasma collected day 0 while the day 3 sample had negligible amounts close to that of healthy controls (Fig 13D). In order to study the *IL-1β* response in an experimental model, we utilized a three dimensional (3D) tissue model of human skin [29], which was infected with GAS strain 2006. Gram staining of infected tissue models revealed bacterial dissemination throughout the entire tissue already 24h after infection and similarly to the patient findings, high bacterial load was evident at 48h after infection (Fig 13E). Furthermore, analysis of tissue model supernatants revealed increasing IL-1β levels over the infection period, whereas in unstimulated models only background levels were detected (Fig 13F).

## Discussion

The ARI panel of BXD mouse strains has been widely used to identify the host genetic loci for pathogenic disease susceptibility [26, 27, 30–33]. The major advantage of using this mouse model in the study of complex host-pathogen interactions is that the genomes of the two ancestral parents of the BXD strains, namely B6 and D2, differ from each other by approximately 1.8 million SNPs, and thus the genome of each fully genotyped, renewable BXD strain has a unique recombination mixture of chromosomal segments inherited from either parent [25, 34]. In this study, we utilized 33 such BXD strains (along with the B6, D2, and B6D2F1 strains) and mapped three quantitative phenotypic traits—survival, weight change, and lesion size, to define the genetic architecture of GAS NSTIs in the mouse. In addition to strain variability (host genetic context), nongenetic cofactors including sex, age, and body weight are also significant predictors in controlling the susceptibility and severity of GAS NSTIs. The major findings of this study include the identification of four QTL on mouse chromosomes (Chr 2, 6, 7, and 18) and the identification of 375 host candidate genes (S1–S4 Tables) that have a likely role in dictating severity, susceptibility, and manifestations of GAS NSTIs. Further differential expression analyses associated interleukin-1 beta pathway as key network involved in regulating GAS NSTIs severity and susceptibility.

In our initial studies with conventional inbred and outbred mouse models [24] and in this study, the parental strain D2 was more susceptible to GAS NSTIs than was the B6 strain, but interestingly the resistant B6 strain lost more weight and developed larger lesions than did D2. Susceptibility, in terms of the three phenotypic traits based on their relative genotypes, differed greatly among the BXD strains. For instance, BXD strains with D haplotypes within the survival QTL on mouse Chr 2 were susceptible to NSTIs compared to BXD strains harboring B haplotypes. Further, BXD strains with D haplotypes within the lesion QTL on Chr 6 and B haplotypes within the weight change QTL on mouse Chr 7 and/or lesion size QTL on Chr 18 lost the most weight and developed large lesions respectively. Based on these findings, we can predict the relative severity and/or manifestations of GAS NSTIs in any of the BXD strains. For instance, BXD 40 was an extremely susceptible strain in our study; retrospectively, we associated susceptibility and severity with the presence of B haplotypes within the QTL on Chr 7 (increased weight loss) and on Chr 18 (large lesions) and the D haplotypes on the QTL on Chr 6 (large lesions) and Chr 2 (increased mortality, bacterial load, and dissemination). We propose the collective polygenic contributions of these four QTL enabled BXD 40 to be more susceptible than the D2 parent. In contrast, like the resistant B6 parents, BXD 73b and 87 strains harbor B haplotypes on the Chr 18 QTL (lesion size) and on the Chr 2 QTL (increased survival with reduced bacterial load and dissemination); therefore, these strains are resistant to GAS NSTIs and recover if such infection occurs. However, the additional D haplotypes on Chr 6 QTL (large lesions) may be associated with both the BXD strains developing larger lesions, whereas the incomplete association of B haplotypes on proximal Chr 7 QTL (increased weight loss) may be the reason why BXD 87 lost less weight than the B6 parental strain did.

Our prior studies using the BXD strains to define the genetic susceptibility loci for survival against GAS sepsis mapped to three QTL, including a strong QTL between 24 and 34 Mb on mouse Chr 2 that had a peak LRS of 34.2 (GN trait ID: 10836) [30]. Although both studies were independent and involved different forms of invasive M1T1 GAS isolates (5448WT for NSTIs vs. 5448AP for sepsis), which were administered by two different routes (subcutaneous vs. intravenous tail vein injections), we located one overlapping QTL on mouse Chr 2 for survival in both studies. In fact, 5448AP is an isogenic, animal-passaged variant of the clinical isolate 5448WT, which exhibits a hypervirulent phenotype and has an expression profile of virulence factors that differs from that of its parental strain. The main advantage of 5448AP is that it lacks the cysteine protease SpeB, which can degrade host and pathogenic proteins without discrimination, and in the absence of SpeB, 5448AP preserves most of its virulent signature proteins and thereby evades the host immune strategies [35–39]. Host signals including those of neutrophils [40], transferrin and lactoferrin [41], and transition metals (zinc and copper) [42] have been shown to facilitate the development of a SpeB-negative phenotype in 5448WT. However, SpeB is needed for skin infections [43], and that was one of the reasons we used 5448WT for our GAS NSTIs studies. Taken together, we speculate that the 5448WT strain produces SpeB needed for initial NSTI pathogenesis, but once the pathogen reaches deep layers of skin tissue and enters the bloodstream, it may become a hypervirulent variant (5448AP) that causes sepsis in addition to NSTIs. For this reason, we believe the two independent BXD studies (sepsis and NSTIs) studying the genetic susceptibility loci for survival showed an overlap in QTL on mouse Chr 2. We are currently exploring this scenario in depth to understand the exact spatio-temporal transition from WT to AP phenotype and the molecular host-pathogen interactions that lead to this transition. In addition, we are determining whether host genetics has a role in influencing this transition by mapping the transition ratio (ratio of SpeB-negative phenotypes in the total bacteria isolated from the infected skin and/or organs of each BXD mouse at specific time points after infection) as a quantitative phenotype. In this way, we will define the QTL encompassing the candidate genes influencing this transition.

We used the QTLminer tool to explore the four mapped host susceptibility loci and extracted 375 rank-ordered, biologically relevant host candidate genes, most of which are polymorphic in both ancestral strains (B6 and D2) and are involved in inflammation, innate immunity, cell cycle, and apoptosis among other biological processes (S1–S4 Tables). Our differential expression analyses of these candidate genes between the representative resistant (BXD 73 and 87) and susceptible (BXD 40 and 64) BXD strains before and after GAS NSTIs further narrowed the list of candidate genes. Specifically, 125 genes were significantly differentially expressed in susceptible strains compared to their uninfected controls (S5–S7 Tables). Significantly down regulated genes on mouse Chr 2 (trait: survival) and Chr 6 (trait: lesion size) included abelson murine leukemia oncogene 1 (*Abl1*), adenylate kinase 1 (*Ak1*), anaphase promoting complex subunit 2 (*Anapc2*), endothelial differentiation-related factor 1 (*Edf1*), ets variant gene 6 (*Etv6*), LIM homeobox transcription factor 1 beta (*Lmx1b*), netrin G2 (*Ntng2*), nucleoporin 214 (*Nup214*), notch gene homolog 1 (*Notch1*), retinoid X receptor alpha (*Rxra*) and tuberous sclerosis 1 (*Tsc1*); each is involved in cell cycle arrest, cell proliferation and differentiation, angiogenesis, and apoptosis as indicated by the gene ontology (GO) annotation data provided by QTLminer (S1–S7 Tables). Further studies are needed to elucidate if and how these observed quantitative differences relate to differential susceptibility to GAS NSTIs. On the other hand, expression of the gene for glycerophosphodiester phosphodiesterase 1 (*Gde1*) on Chr 7 (trait: weight change) has been associated with triglyceride accumulation in mice [44] and in our case *Gde1* was significantly down regulated in susceptible strains compared to their uninfected controls. We hypothesize that this may be one of the reasons for the observed weight loss in these two susceptible BXD strains (Figs 3 and 7).

Our gene network analyses of these 125 genes further identified two high-scoring gene networks that likely play a significant role in determining susceptibility to GAS NSTIs, particularly highlighting interleukin-1 β (*IL-1β*) as key regulator likely to participate in modulating GAS NSTIs (Fig 11). *IL-1β* produced chiefly by monocytes, macrophages, and dendritic cells, belongs to the interleukin-1 family of cytokines, and is the highly inflammatory cytokine associated with several inflammatory conditions [45]. Indeed, our subsequent gene expression analyses revealed us that *IL-1β* was in fact significantly up regulated in the susceptible D2 parental strains compared to B6; also, there was a significant correlation between infection severity (as measured by survival index) and *IL-1β* expression levels (Fig 12). We also observed *IL-1β* to be significantly up regulated in patient biopsies as compared to biopsies from healthy controls (Fig 13C). Besides, the protein levels of IL-1β were also increased in patient plasma samples and in infected skin models compared to their respective controls (Fig 13D and 13F), suggesting that GAS NSTIs susceptibility is associated with an amplified inflammatory response mediated by *IL-1β*. However, although *IL-1β*-dependent macrophage immune response has been shown to be protective in an intravenous mouse model of GAS sepsis utilizing 5448AP bacteria [46], female CD-1 strains were used for these infections and we have shown that these strains are resistant to GAS NSTIs mediated by 5448WT bacteria [24]. Moreover, similar to what we mentioned earlier, both these studies use two different infection models (sepsis vs. NSTIs) and bacteria (5448AP vs. 5448WT). Taken together, we believe that host genetics- and context-dependent differential regulation of *IL-1β* can modulate the severity and mortality of GAS invasive infections. Further investigations are warranted to have a better understanding of this phenomenon.

In conclusion, our unbiased forward systems genetics approaches utilizing the ARI panel of BXD mice strains has defined the genetic architecture of GAS NSTIs in mice revealing four NSTIs-associated QTL on mouse Chr 2, 6, 7 and 18, and has allowed us to extract 375 host candidate genes. Additional differential expression analyses between the representative resistant (BXD 73 and 87) and susceptible (BXD 40 and 64) BXD strains, before and after GAS NSTIs, allowed us to identify 125 significantly differentially expressed genes in susceptible strains compared to their uninfected controls. Our IPA analyses of these 125 genes underscored *IL-1β* as potentially a key regulator of GAS NSTIs susceptibility; a finding that was also supported by its up regulation in patient tissue biopsies as well as in GAS infected experimental models including mice and engineered 3D human skin tissue. Our ongoing in-depth pathway analyses to identify additional molecular interactions between all the differentially expressed genes will help us dissect various host mechanisms and/or interactions with GAS resulting in the differential susceptibility to NSTIs.

## Materials and Methods

### Ethics statement

#### Mice

All animal experiments were carried out in accordance with the recommendations in the Guide for the Care and Use of Laboratory Animals of the National Institutes of Health and with the prior approval of the Institutional Animal Care and Use Committees (IACUC) of the University of Cincinnati (UC approval number 08-03-28-02) and University of North Dakota (UND approval number 1310–01).

#### Patient material and samples from healthy controls

All studies were conducted in accordance with the Helsinki Declaration. All experiments involving human tissue biopsies and plasma samples were approved by the regional Ethical Review Board at the National Committee on Health Research Ethics in Copenhagen (Ref No.: 1211709), Ethical Research Committee at Huddinge University Hospital (Forskningskommitté Syd) (Reference No.: 360/00) and the regional Ethics Committee in Stockholm (Ref. No.: 2006/231-31/4 and 2012/2110-31/2). Written informed consent was obtained from all individuals from who skin tissue biopsies were collected and provided anonymously. As the plasma donors were individuals well acquainted with the research conducted; verbal informed consent was deemed sufficient.

### Mice

For the NSTI studies, we used the ARI lines (BXD) [25], their ancestral parental strains (C57BL/6J [B6] and DBA/2J [D2]), and their F1 population (B6D2F1). All mouse strains were obtained from the in-house breeding colonies at UC and UND. A total of 629 mice were used; 508 mice (262 males and 246 females), after the exclusions based on previously described, predetermined criteria [30, 31] were considered for final analyses.

### Patient material and samples from healthy controls

Tissue biopsies and plasma samples collected from one GAS NSTI patient, a 61 year old male (patient ID 2006), enrolled at Rigshospitalet in Copenhagen as part of the EU-funded project INFECT (www.fp7infect.eu) were analyzed. The tissue biopsies were collected from the site of infection, e.g. fascia (day 1) and soft tissue (day 2), during surgical procedures and immediately snap-frozen. Skin tissue from healthy controls was obtained at plastic surgery at the Karolinska University Hospital. Also, plasma samples were obtained from healthy individuals.

### Bacteria and culture media

We used a representative M1T1 clonal GAS isolate 5448WT (for animal studies) and NSTI patient M1 GAS isolate 2006 (for skin model studies), which were routinely grown at 37°C in THY medium (Todd-Hewitt broth (Difco) supplemented with 1.5% (w/v) yeast extract) statically as described previously [10].

### Eukaryotic cells and culture conditions

The human keratinocyte cells (N/TERT-1) were maintained in EpiLife medium (Invitrogen). Normal human dermal fibroblasts were cultured in DMEM (Invitrogen) supplemented with 10% (v/v) fetal bovine serum (FBS; Invitrogen). Both were cultured at 37°C under a 5% CO_2_ atmosphere.

### 3D organotypic skin-model and infection

The models were generated following the protocol as previously published [29] and were infected with 1 x 10^6^ CFU of M1 GAS isolate 2006 for 24 and 48h.

### NSTIs studies and post-infection monitoring

Hair was removed from the dorsal side of the mouse with Nair hair removal lotion (Church & Dwight Co., Inc., Ewing, NJ) one day prior to infection. Groups of 5–36 mice from a total of 33 BXD strains, their ancestral parental strains (B6 and D2), and the B6D2F1 strains were infected subcutaneously under the skin with 1 x 10^8^ CFU of 5448WT per mouse (bacteria were suspended in 100 μL of sterile phosphate-buffered saline [PBS] per dose), while control animals were given injections of sterile PBS alone. After inoculation, each mouse was housed in individual cages to avoid contact/influence from other animals over lesions. Animals were observed twice a day for the next seven days for mortality, weight change, and lesions. For bacterial load estimation studies, animals were humanely sacrificed (euthanized) if needed during the seven-day infection timeline (or at the experimental endpoint). Blood was drawn through cardiac puncture for bacteremia estimations; necrotic skin tissues and spleens were also recovered and homogenized by a rotor stator homogenizer (Omni International, Marietta, GA) for enumeration to determine bacterial load and dissemination, respectively.

### Phenotypic trait analyses

#### Trait 1: Survival

Mean values of corrected relative survival indices (cRSI) for each strain were assigned as described [30, 31]. Briefly, multimodal distribution clusters of survival days were converted into a normalized survival index and assigned to each mouse irrespective of its strain. Finally, these indices were corrected for significant covariates, including mouse strain, age, sex, and body weight, by performing a general linear model (GLM) analysis using ordinary least squares analysis of variance (OLS ANOVA).

#### Trait 2: Weight change kinetics

Following a similar method of GLM analysis, we determined mean values of corrected percent weight change data for each strain for days 1–4. Principal component analysis (PCA) was done to combine the four non-independent data.

#### Trait 3: Lesion size

Using a similar method of GLM analysis, we determined mean values of corrected maximum lesion area data for each strain.

### Heritability calculation

We calculated broad sense heritability as previously described with slight modifications [27, 31]. We used the OLS ANOVA table computed by GLM analysis to calculate broad sense heritability, which was expressed as the ratio of genetic variance (mean square of strain) to total variance (sum of mean square of strain and residuals).

### Genome-wide linkage scans and mining for host candidate genes

We performed genome-wide linkage scans (GWLS) to identify QTL by using a web-based QTL/interval mapping tool (WebQTL) available on the Gene Network (GN) website (www.genenetwork.org). This tool scans the mouse genome for QTLs and estimates the strength of the linkage as likelihood ratio statistic (LRS) using 5000 permutation tests [34]. We performed three sets of GWLS by using strain means for the following three quantitative phenotypic variables, for which the original datasets can be obtained at GN by using the associated identification numbers: (i) corrected relative survival index (GN Trait ID: 17524), (ii) corrected percent weight change kinetics for days 1–4 and their principal component (PC1) (GN Trait ID: 17520–17523 and 17527), and (iii) corrected maximum lesion area (GN Trait ID: 17525). We used the web-based QTLminer tool available in the GN website to rank-order and shortlist the host candidate genes on the mapped QTL [28].

### Quantitative real-time PCR array analyses

Representative BXD strains selected based on their susceptibility to GAS NSTIs (BXD 40 and 64 for susceptible strains, and BXD 73 and 87 for resistant strains) was subcutaneously infected with either 1x10^8^ GAS 5448WT or mock infected with PBS. Three independent experiments (n ≥ 3 mouse per strain, per experiment) were performed. Forty-eight hours after infection, animals were humanely sacrificed (euthanized) and necrotic skin tissues were excised from each of the mouse, from which total RNA was isolated using FastRNA PRO green kit (MP Biomedicals, LLC, Santa Ana, CA), and then purified using GeneJET RNA cleanup and concentration micro kit (ThermoFisher Scientific, Grand Island, NY). RNA samples (with A260/280 ≥ 1.9) from same strain and sex were pooled together for cDNA synthesis by SensiFAST cDNA synthesis kit (Bioline, Taunton, MA). We custom designed 384-well real-time PCR plates with 384 unique PrimePCR SYBR Green assays (Bio-Rad, Hercules, CA) with most gene specific primers that span long introns to distinguish cDNA from genomic DNA and utilized Bio-Rad CFX384 Touch real-time PCR detection system (Bio-Rad, Hercules, CA). We used beta actin (*Actb*), beta glucuronidase (*Gusb*) and ribosomal protein L7A (*Rpl7a*) as the endogenous control, which were used to normalize our gene expression data.

Real-time PCR experiments often results in non-detects, where the PCR reactions failed to produce a minimum amount of signal due to various reasons. Studies have shown that imputing missing expression values contribute to less biased estimation of non-detects from real-time PCR experiments [47, 48]. The PrimePCR analysis software (Bio-Rad, Hercules, CA), used in this study to obtain relative normalized fold regulation and statistical significance (*P* values) by using delta delta Cq (quantification cycle) method, does not perform a statistical evaluation for differential expression for those genes with any number of non-detects. Therefore, in order to maximize the identification of differentially expressed genes, the non-detects in our real-time PCR results were imputed using the ImputeMissingValuesKNN module available in the GenePattern server (The Broad Institute; http://www.broadinstitute.org). The imputation was limited to those genes with one non-detect among the eight samples. False discovery rates (FDR) were computed using p.adjust function in R statistics library (http://cran.r-project.org/).

For individual mouse real-time PCR reactions of interleukin-1 beta (*IL-1β*), we used 5’-CACAGCAGCACATCAACAAG-3’ (forward) and 5’- GTGCTCATGTCCTCATCCTG-3’ (reverse) as primer pairs. Beta actin (*Actb*) with primer pairs, 5’-ATGGAGGGGAATACAGCCC-3’ (forward) and 5’-TTCTTTGCAGCTCCTTCGTT-3’ (reverse), was used as the endogenous control to normalize *IL-1β* gene expression data. We manually computed relative normalized expression using Livak’s method [49], and *P* values using student’s t-test.

From patient biopsies and healthy controls, total RNA was isolated using RiboPure RNA purification Kit (Ambion) according to manufacturer’s guidelines. cDNA synthesis was performed using the Superscript first-strand synthesis system for RT-PCR (Invitrogen). The following primer sets were used: hbetaAct_F: 5′- CTCTTCCAGCCTTCCTTCCT-3′, hbetaAct_R: 5′- AGCACTGTGTTGGCGTACAG-3′, hIL-1β_F: 5′-GCCCTAAACAGATGAAGTGCTC-3′, hIL-1β_R: 5′- GAACCAGCATCTTCCTCAG-3′. The real-time PCR amplification was performed with SYBR GreenER Kit (Invitrogen) using an ABI Prism 7500 sequence detection system (Applied Biosystems). The levels of β-actin transcription were used for normalization.

### Ingenuity pathway analyses

We used QIAGEN’s Ingenuity Pathway Analysis (IPA, QIAGEN Redwood City, http://www.qiagen.com/ingenuity) to generate gene networks within the 125 significantly differentially regulated genes by uploading them along with their fold regulation and FDR values into the online application, and we chose the top 2 significant networks. Gene networks were generated based on their connectivity and the significance was measured in two ways: (1) the ratio of the number of genes from the dataset that map to the pathway divided by the total number of genes that map to the pathway; and (2) by Fischer’s exact test with *P* < 0.001.

### Gram staining of patient biopsies and skin tissue

For cryosectioning, skin tissue models were pretreated with 2M sucrose for 1h before embedding in optimum cutting temperature compound (Sakura Finetek) followed by freezing in liquid nitrogen and stored at -80°C. Snap frozen patient biopsies were embedded without pretreatment. 8μm cryosections were obtained using a MICROM cryostat HM 560 MV (Carl Zeiss) and fixed in 2% freshly prepared formaldehyde in PBS for 15 minutes at room temperature. Bacteria were visualized *via* conventional Gram staining.

### IL-1β ELISA

The levels of IL-1β in supernatants of infected skin tissue models, as well as plasma samples from patient 2006 and healthy controls were measured using human IL-1β Quantikine ELISA (R&D Systems) according to manufacturer’s guidelines. The range of detection was 0–2000 pg/ml.

### Statistical analyses

Multimodal distribution clusters, mean survival indices, corrected survival indices, corrected percent weight change, corrected maximum lesion area, GLM analyses, and heritability were calculated by DataDesk 6.3 (Data Description, Inc., Ithaca New York USA, www.datadesk.com) as described previously [30, 31]. Principal component analyses (PCA) and false discovery rates (FDR) were computed using R statistical analysis software version 3.2.1 (http://cran.r-project.org/). Other statistical analyses were performed with Prism v6.0d (GraphPad Software, La Jolla California USA, www.graphpad.com). Survival analyses were done using the log-rank (Mantel-Cox) test. One-way analysis of variance (ANOVA) was used to evaluate differences in log-transformed bacterial loads (skin, blood and spleens) between the different mouse strains. We set the critical significance value (α) at 0.05, and if the *P* values were less than α, we reported that the observed differences were statistically significant.

## Supporting Information

S1 Table Host candidate genes in the mapped QTL for survival (GN trait ID 17524) on mouse Chr 2 between 24.5 and 35Mb. (PDF)

S2 Table Host candidate genes in the mapped QTL for percent weight change, PC1 (GN trait ID: 17527) on mouse Chr 7 between 125 and 131Mb. (PDF)

S3 Table Host candidate genes in the mapped QTL for lesion size (GN trait ID: 17525) on mouse Chr 6 between 131.6 and 141.8Mb. (PDF)

S4 Table Host candidate genes in the mapped QTL for lesion size (GN trait ID: 17525) on mouse Chr 18 between 49.5 and 56.3Mb. (PDF)

S5 Table Relative normalized expression levels of host candidate genes for survival (GN trait ID 17524) on mouse Chr 2, after infection in susceptible BXD strains. (PDF)

S6 Table Relative normalized expression levels of host candidate genes for percent weight change, PC1 (GN trait ID: 17527) on mouse Chr 7, after infection in susceptible BXD strains. (PDF)

S7 Table Relative normalized expression levels of host candidate genes for lesion size (GN trait ID: 17525) on mouse Chr 6 and 18, after infection in susceptible BXD strains. (PDF)

S8 Table List of genes in the functional gene network given by IPA. (PDF)
