# Supplementary material for: Genetic Architecture of Group A Streptococcal Necrotizing Soft Tissue Infections in the Mouse
Source: PLoS Pathog. 2016 Jul 11;12(7):e1005732. doi: 10.1371/journal.ppat.1005732 (PMC4939974; doi:10.1371/journal.ppat.1005732)
Supplement: S1 Table — (PDF) [file ppat.1005732.s001.pdf]

S1 Table. Host candidate genes in the mapped QTL for survival (GN trait ID 17524) on mouse Chr 2 between 24.5 and 35Mb

Gene symbol	Chr 2 (Mb)	Gene description	GO biological process	nsSNPs (B6 vs. D2)	Indels in BXD	Score (0-4)
Traf1	34.8	Tnf receptor-associated factor 1	Apoptosis, regulation of apoptosis, signal transduction	26	3	4
St6galnac6	32.46	ST6 (alpha-N-acetyl-neuraminyl-2,3-beta-galactosyl-1,3)-N-acetylgalactosaminide alpha-2,6-sialyltransferase 6	Protein amino acid glycosylation, ganglioside biosynthetic process	35	4	4
Ptges	30.74	Prostaglandin E synthase	Prostaglandin biosynthetic process, negative regulation of cell proliferation	4	1	4
Agpat2	26.45	1-acylglycerol-3-phosphate O-acyltransferase 2 (lysophosphatidic acid acyltransferase, beta)	Phospholipid biosynthetic process, metabolic process	2	4	4
Notch1	26.31	Notch gene homolog 1 (Drosophila)	Keratinocyte differentiation, Notch signaling pathway, positive regulation of cell proliferation	15	7	4
Entpd2	25.25	Ectonucleoside triphosphate diphosphohydrolase 2	G-protein coupled receptor protein signaling pathway, platelet activation	21	2	4
Ehmt1	24.65	Euchromatic histone methyltransferase 1	Negative regulation of transcription from RNA polymerase II promoter	15	15	4
Gapvd1	34.53	GTPase activating protein and VPS9 domains 1	Endocytosis, regulation of small GTPase mediated signal	6	1	4

			transduction, regulation of protein transport			
Mapkap1	34.29	Mitogen-activated protein kinase associated protein 1	Response to stress	19	11	4
Ralgps1	32.99	Ral GEF with PH domain and SH3 binding motif 1	Small GTPase mediated signal transduction, biological process	6	1	4
Pnpla7	24.83	Patatin-like phospholipase domain containing 7	Lipid catabolic process, lipid metabolic process, metabolic process	38	6	4
Dph7	24.82	Diphthamine biosynthesis 7	Biological process	30	5	4
Zmynd19	24.81	Zinc finger, MYND domain containing 19	Biological process	4	4	4
Arrdc1	24.78	Arrestin domain containing 1	Biological process	15	2	4
Uap1l1	25.22	UDP-N-acteylglucosamine pyrophosphorylase 1-like 1	Metabolic process, biological process	34	2	4
Mrpl41	24.83	Mitochondrial ribosomal protein L41	Cell cycle, apoptosis, biological process	16	1	4
Egfl7	26.44	EGF-like domain 7	Cell differentiation, angiogenesis, multicellular organismal development	32	2	4
Npdc1	25.26	Neural proliferation, differentiation and control gene 1		8	2	4
Hmcn2	31.17	Hemicentin 2		101	27	4
Hc	34.84	Hemolytic complement	Cytolysis, innate immune response, complement activation, leukocyte migration during inflammatory response	62	4	3

St6galnac4	32.44	ST6 (alpha-N-acetyl-neuraminyl-2,3-beta-galactosyl-1,3)-N-acetylgalactosaminide alpha-2,6-sialyltransferase 4	Protein amino acid glycosylation	3	0	3
Abca2	25.28	ATP-binding cassette, sub-family A (ABC1), member 2	Transport, cholesterol metabolic process	6	2	3
Crat	30.26	Carnitine acetyltransferase	Transport, lipid metabolic process, fatty acid metabolic process	9	0	3
Fut7	25.28	Fucosyltransferase 7	Protein amino acid glycosylation	5	2	3
Dnm1	32.16	Dynamin 1	Endocytosis, receptor-mediated endocytosis	3	1	3
Cacna1b	24.46	Calcium channel, voltage-dependent, N type, alpha 1B subunit	Transport, ion transport, transmembrane transport, positive regulation of neurotransmitter secretion	50	27	3
Pbx3	34.03	Pre B-cell leukemia transcription factor 3	Regulation of transcription, DNA-dependent, regulation of respiratory gaseous exchange by neurological system process	4	1	3
Lrsam1	32.78	Leucine rich repeat and sterile alpha motif containing 1	Protein transport, transport, biological process	3	1	3
Gpr107	31.01	G protein-coupled receptor 107	Biological process	39	12	3
Usp20	30.85	Ubiquitin specific peptidase 20	Endocytosis, ubiquitin-dependent protein catabolic process	3	0	3
Sec16a	26.26	SEC16 homolog A (S. cerevisiae)	Positive regulation of neuron projection development	18	1	3

Snapc4	26.22	Small nuclear RNA activating complex, polypeptide 4	Transcription, regulation of transcription, biological process	26	2	3
Qsox2	26.06	Quiescin Q6 sulfhydryl oxidase 2	Cell redox homeostasis, biological process, oxidation reduction	26	2	3
Kcnt1	25.72	Potassium channel, subfamily T, member 1	Transport, ion transport, metabolic process, potassium ion transport	49	8	3
BC029214	25.32	cDNA sequence BC029214	Biological process	8	0	3
Clic3	25.31	Chloride intracellular channel 3	Transport, ion transport, chloride transport, biological process	7	1	3
Dpp7	25.21	Dipeptidylpeptidase 7	Proteolysis	6	0	3
Fnbp1	30.88	Formin binding protein 1	Endocytosis, nervous system development	2	1	3
Lcn11	25.63	Lipocalin 11	Biological process	9	1	3
Ndor1	25.1	NADPH dependent diflavin oxidoreductase 1	Oxidation reduction, biological process	3	0	3
Mamdc4	25.42	MAM domain containing 4	Biological process	3	1	3
Lmx1b	33.42	LIM homeobox transcription factor 1 beta	Regulation of transcription, cell death, cell proliferation, organ growth	5	4	3
Ppp2r4	30.27	Protein phosphatase 2A, regulatory subunit B (PR 53)		65	9	3
Rabl6	25.44	RAB, member RAS oncogene family-like 6	Small GTPase mediated signal transduction	11	1	3
Sapcd2	25.23	Suppressor APC domain containing 2	Biological process	20	2	3
Fpgs	32.54	Folylpolyglutamyl	One-carbon	3	0	2

		synthetase	metabolic process, folic acid and derivative biosynthetic process			
Ak1	32.49	Adenylate kinase 1	Cell cycle arrest, nucleobase, nucleoside, nucleotide and nucleic acid metabolic process	3	0	2
Ptges2	32.25	Prostaglandin E synthase 2	Fatty acid biosynthetic process, prostaglandin biosynthetic process	3	0	2
Sh3glb2	30.2	SH3-domain GRB2-like endophilin B2	Biological process	1	0	2
Slc27a4	29.66	Solute carrier family 27 (fatty acid transporter), member 4	Fatty acid metabolic process, transport, lipid metabolic process, lipid transport	2	0	2
Col5a1	27.74	Collagen, type V, alpha 1	Cell adhesion, blood vessel development, skin development	1	0	2
Abo	26.7	ABO blood group (transferase A, alpha 1-3-N-acetylgalactosaminyltransferase, transferase B, alpha 1-3-galactosyltransferase)	Positive regulation of cell proliferation, negative regulation of apoptosis, cellular oligosaccharide metabolic process	3	0	2
Traf2	25.37	Tnf receptor-associated factor 2	Regulation of apoptosis, tumor necrosis factor-mediated signaling pathway	11	0	2
C8g	25.35	Complement component 8, gamma subunit	Cytolysis, innate immune response, complement activation	11	0	2

Man1b1	25.19	Mannosidase, alpha, class 1B, member 1	ER-associated protein catabolic process	2	0	2
Anapc2	25.13	Anaphase promoting complex subunit 2	Mitosis, cell cycle, cell division, ubiquitin-dependent protein catabolic process	2	0	2
Ptgds	25.32	Prostaglandin D2 synthase (brain)	Fatty acid biosynthetic process, transport, prostaglandin biosynthetic process	17	0	2
Uck1	32.11	Uridine-cytidine kinase 1		1	0	2
Zbtb43	33.31	Zinc finger and BTB domain containing 43	Transcription, regulation of transcription, biological process	6	0	2
Garnl3	32.84	GTPase activating RANGAP domain-like 3	Regulation of small GTPase mediated signal transduction, biological process	5	0	2
Eng	32.5	Endoglin	Heart development, angiogenesis, regulation of transforming growth factor beta receptor signaling pathway	0	1	2
BC005624	30.83	cDNA sequence BC005624	Biological process	0	1	2
Asb6	30.68	Ankyrin repeat and SOCS box-containing protein 6	Biological process, intracellular signaling cascade	3	0	2
Lrrc8a	30.09	Leucine rich repeat containing 8A	Biological process	2	0	2
Pkn3	29.93	Protein kinase N3	Protein amino acid phosphorylation, signal	0	0	2

			transduction, biological process			
Setx	28.98	Senataxin	DNA repair, response to DNA damage stimulus, biological process	1	0	2
Ttf1	28.92	Transcription termination factor 1	Negative regulation of DNA replication, transcription	1	0	2
Gfi1b	28.46	Growth factor independent 1B	Transcription, regulation of transcription, multicellular organismal development	1	0	2
Rexo4	26.81	REX4, RNA exonuclease 4 homolog (S. cerevisiae)	Biological process	2	0	2
4932418E24Rik	26.13	RIKEN cDNA 4932418E24 gene	Biological process	3	0	2
Ubac1	25.85	Ubiquitin associated domain containing 1	Biological process	0	3	2
Lcn10	25.54	Lipocalin 10	Transport, biological process	0	1	2
Edf1	25.41	Endothelial differentiation-related factor 1	Cell differentiation, transcription, multicellular organismal development	5	0	2
Fbxw5	25.36	F-box and WD-40 domain protein 5	Biological process	6	0	2
Lcn12	25.35	Lipocalin 12	Transport, biological process	17	0	2
Rnf208	25.1	Ring finger protein 208	Biological process	1	0	2
Slc34a3	25.08	Solute carrier family 34 (sodium phosphate), member 3	Phosphate transport, sodium ion transport	3	0	2
Stxbp1	32.64	Syntaxin binding protein 1	Protein transport, synaptic vesicle maturation, axon target recognition	3	0	2

Olfm1	28.05	Olfactomedin 1	Multicellular organismal development, protein oligomerization	1	0	2
Med22	26.76	Mediator complex subunit 22	Transcription, regulation of transcription	3	0	2
Lcn5	25.51	Lipocalin 5	Transport, retinoic acid metabolic process, lipid metabolic process	3	0	2
Gm996	25.43	Predicted gene 996	Biological process	8	0	2
Camsap1	25.76	Calmodulin regulated spectrin-associated protein 1	Biological process	0	1	2
Lhx3	26.06	LIM homeobox protein 3	Regulation of transcription, negative regulation of apoptosis, cell differentiation	2	0	2
Fbxw2	34.66	F-box and WD-40 domain protein 2		2	0	2
Phyhd1	30.12	Phytanoyl-CoA dioxygenase domain containing 1		0	1	2
Surf4	26.78	Surfeit gene 4		3	0	2
Mvb12b	33.59	Multivesicular body subunit 12B	Protein transport, transport	26	19	2
Ncs1	31.1	Neuronal calcium sensor 1	Positive regulation of synaptic transmission, positive regulation of exocytosis	10	8	2
Ntmt1	30.66	N-terminal Xaa-Pro-Lys N-methyltransferase 1	Biological process	6	1	2
Nacc2	25.91	Nucleus accumbens associated 2, BEN and BTB (POZ) domain containing	Biological process	14	3	2

Tprn	25.12	Taperin	Biological process	5	1	2
Hspa5	34.63	Heat shock protein 5	ER overload response, negative regulation of transforming growth factor beta receptor signaling pathway	0	0	1
Dpm2	32.43	Dolichol-phosphate (beta-D) mannosyltransferase 2	GPI anchor biosynthetic process, dolichol metabolic process	0	0	1
Pomt1	32.09	Protein-O-mannosyltransferase 1	Extracellular matrix organization, protein amino acid O-linked glycosylation	0	0	1
Lamc3	31.74	Laminin gamma 3	Cell adhesion	0	0	1
Exosc2	31.53	Exosome component 2	rRNA processing, biological process	0	0	1
Ass1	31.33	Argininosuccinate synthetase 1	Arginine biosynthetic process, response to zinc ion	0	0	1
Dolpp1	30.25	Dolichyl pyrophosphate phosphatase 1	Protein amino acid N-linked glycosylation	0	0	1
Ccbl1	30.04	Cysteine conjugate-beta lyase 1	Biosynthetic process, biological process	0	0	1
Endog	30.03	Endonuclease G	Positive regulation of apoptosis, response to tumor necrosis factor	0	0	1
Tsc1	28.5	Tuberous sclerosis 1	Negative regulation of cell proliferation, negative regulation of cell size, regulation of protein kinase activity	0	0	1

Ralgds	28.37	Ral guanine nucleotide dissociation stimulator	Regulation of small GTPase mediated signal transduction	0	0	1
Vav2	27.12	Vav 2 oncogene	Angiogenesis, regulation of Rho protein signal transduction, cell migration	0	0	1
Sardh	27.04	Sarcosine dehydrogenase	Oxidation reduction, glycine catabolic process, biological process	0	0	1
Rpl7a	26.77	Ribosomal protein L7a	Ribosome biogenesis, biological process	0	0	1
Inpp5e	26.25	Inositol polyphosphate-5-phosphatase E	Lipid metabolic process, phosphoinositide metabolic process	0	0	1
Phpt1	25.43	Phosphohistidine phosphatase 1	Biological process	0	0	1
Grin1	25.15	Glutamate receptor, ionotropic, NMDA1 (zeta 1)	Suckling behavior, male mating behavior, regulation of neuron apoptosis	0	0	1
Rapgef1	29.48	Rap guanine nucleotide exchange factor (GEF) 1	Cell-cell adhesion, blood vessel development, platelet-derived growth factor receptor signaling pathway	0	0	1
Rxra	27.53	Retinoid X receptor alpha	Positive regulation of transcription from RNA polymerase II promoter, cardiac muscle cell proliferation	0	0	1
Abl1	31.54	v-abl Abelson murine leukemia oncogene 1	Peptidyl-tyrosine phosphorylation, regulation of cell cycle, positive	0	0	1

			regulation of apoptosis			
Gbgt1	28.35	Globoside alpha-1, 3-N-acetylgalactosaminyltransferase 1	Carbohydrate metabolic process, lipid glycosylation	0	0	1
Entpd8	24.94	Ectonucleoside triphosphate diphosphohydrolase 8	Nucleoside diphosphate biosynthetic process, nucleoside monophosphate biosynthetic process	0	0	1
Dbh	27.02	Dopamine beta hydroxylase	Oxidation reduction, leukocyte mediated immunity, regulation of cell proliferation	0	0	1
Card9	26.21	Caspase recruitment domain family, member 9	Regulation of interleukin-6 biosynthetic process, I-kappaB kinase/NF-kappaB cascade, positive regulation of innate immune response	0	0	1
Phf19	34.75	PHD finger protein 19	Biological process, regulation of transcription	0	0	1
Psmd5	34.71	Proteasome (prosome, macropain) 26S subunit, non-ATPase, 5	Biological process	0	0	1
Angptl2	33.07	Angiopoietin-like 2	Signal transduction	0	0	1
Slc2a8	32.83	Solute carrier family 2, (facilitated glucose transporter), member 8	Transmembrane transport, response to hypoxia, insulin receptor signaling	0	0	1

			pathway			
Ttc16	32.62	Tetratricopeptide repeat domain 16	Biological process	0	0	1
Tor2a	32.61	Torsin family 2, member A	Chaperone mediated protein folding requiring cofactor, biological process	0	0	1
Sh2d3c	32.58	SH2 domain containing 3C	Small GTPase mediated signal transduction, signal transduction	0	0	1
Pip5kl1	32.43	Phosphatidylinositol-4-phosphate 5-kinase-like 1	Phosphatidylinositol metabolic process, biological process	0	0	1
Slc25a25	32.27	Solute carrier family 25 (mitochondrial carrier, phosphate carrier), member 25	Transport, transmembrane transport, biological process	0	0	1
Lcn2	32.24	Lipocalin 2	Positive regulation of gene expression, response to drug, cellular response to hydrogen peroxide	0	0	1
1110008P14Rik	32.23	RIKEN cDNA 1110008P14 gene	Biological process	0	0	1
Ciz1	32.22	CDKN1A interacting zinc finger protein 1	Positive regulation of DNA replication initiation	0	0	1
Ppapdc3	31.95	Phosphatidic acid phosphatase type 2 domain containing 3	Biological process	0	0	1
Fibcd1	31.67	Fibrinogen C domain containing 1	Signal transduction, biological process	0	0	1
Qrfp	31.66	Pyroglutamylated RFamide peptide	Neuropeptide signaling pathway, positive regulation of blood pressure	0	0	1
Tor1a	30.82	Torsin family 1,	Chaperone	0	0	1

		member A (torsin A)	mediated protein folding requiring cofactor, biological process			
Prrx2	30.7	Paired related homeobox 2	Multicellular organismal development, regulation of transcription	0	0	1
Nup188	30.14	Nucleoporin 188	Transport, protein transport, mRNA transport, transmembrane transport	0	0	1
Dolk	30.14	Dolichol kinase	Biological process	0	0	1
D2Wsu81e	30.03	DNA segment, Chr 2, Wayne State University 81, expressed	Biological process	0	0	1
Tbc1d13	29.99	TBC1 domain family, member 13	Regulation of Rab GTPase activity, biological process	0	0	1
Zer1	29.95	Zer-1 homolog (C. elegans)	Biological process	0	0	1
Zdhhc12	29.95	Zinc finger, DHHC domain containing 12	Biological process	0	0	1
Set	29.92	SET translocation	Nucleosome assembly	0	0	1
Gle1	29.79	GLE1 RNA export mediator (yeast)	Protein transport, mRNA transport, transmembrane transport, poly (A)+ mRNA export from nucleus	0	0	1
Urm1	29.68	Ubiquitin related modifier 1 homolog (S. cerevisiae)	tRNA processing, biological process	0	0	1
Coq4	29.64	Coenzyme Q4 homolog (yeast)	Biological process, ubiquinone biosynthetic process	0	0	1
Trub2	29.63	TruB pseudouridine (psi) synthase	tRNA processing, pseudouridine	0	0	1

		homolog 2 (E. coli)	synthesis, RNA processing, RNA modification			
Med27	29.2	Mediator complex subunit 27	Regulation of transcription from RNA polymerase II promoter	0	0	1
Barhl1	28.76	BarH-like 1 (Drosophila)	Regulation of transcription, DNA-dependent, negative regulation of neuron apoptosis	0	0	1
Ddx31	28.7	DEAD/H (Asp-Glu-Ala-Asp/His) box polypeptide 31	Biological process	0	0	1
Gtf3c4	28.68	General transcription factor IIIC, polypeptide 4	Transcription, biological process	0	0	1
1700026L06Rik	28.55	RIKEN cDNA 1700026L06 gene	Biological process	0	0	1
Gtf3c5	28.42	General transcription factor IIIC, polypeptide 5	Transcription, biological process	0	0	1
Fcnb	27.93	Ficolin B	Innate immune response, complement activation, lectin pathway	0	0	1
Adamtsl2	26.93	ADAMTS-like 2	Biological process	0	0	1
Slc2a6	26.88	Solute carrier family 2 (facilitated glucose transporter), member 6	Transport, carbohydrate transport, biological process	0	0	1
Adamts13	26.83	A disintegrin-like and metallopeptidase (reprolysin type) with thrombospondin type 1 motif, 13	Hemostasis, blood coagulation, proteolysis	0	0	1
Stkld1	26.79	Serine/threonine kinase-like domain containing 1	Protein amino acid phosphorylation, biological process	0	0	1
Surf6	26.75	Surfeit gene 6	Ribosome biogenesis	4	0	1

Pmpca	26.24	Peptidase (mitochondrial processing) alpha	Proteolysis	0	0	1
Gpsm1	26.17	G-protein signalling modulator 1 (AGS3-like, C. elegans)	Cell differentiation, nervous system development, multicellular organismal development, signal transduction	0	0	1
Sohlh1	25.7	Spermatogenesis and oogenesis specific basic helix-loop-helix 1	Regulation of transcription, multicellular organismal development, cell differentiation	0	0	1
Lcn9	25.68	Lipocalin 9	Transport, biological process	0	0	1
Bmyc	25.56	Brain expressed myelocytomatosis oncogene	Regulation of transcription, DNA-dependent	0	0	1
Fcna	25.48	Ficolin A	Signal transduction	0	0	1
Lrrc26	25.15	Leucine rich repeat containing 26	Biological process	0	0	1
Cysrt1	25.09	Cysteine rich tail 1	Biological process	0	0	1
Noxa1	24.94	NADPH oxidase activator 1	Superoxide metabolic process	0	0	1
Tor1b	30.81	Torsin family 1, member B	Chaperone mediated protein folding requiring cofactor, biological process	0	0	1
Cdk9	32.56	Cyclin-dependent kinase 9 (CDC2-related kinase)	Transcription, regulation of transcription, protein amino acid phosphorylation	0	0	1
Fubp3	31.43	Far upstream element (FUSE) binding protein 3	Positive regulation of transcription from RNA polymerase	0	0	1

			II promoter			
Zbtb34	33.26	Zinc finger and BTB domain containing 34	Biological process	0	0	1
Tmem141	25.48	Transmembrane protein 141	Biological process	0	0	1
Sptan1	29.82	Spectrin alpha, non-erythrocytic 1	Actin filament capping, actin cytoskeleton reorganization	0	0	1
Sdccag3	26.24	Serologically defined colon cancer antigen 3	Biological process	0	0	1
Odf2	29.75	Outer dense fiber of sperm tails 2	Cell differentiation, spermatogenesis, multicellular organismal development	0	0	1
Nup214	31.83	Nucleoporin 214	Protein transport, mRNA transport, regulation of cell cycle	0	0	1
Ntng2	29.05	Netrin G2	Cell differentiation, nervous system development, multicellular organismal development	0	0	1
Lcn6	25.53	Lipocalin 6	Biological process	0	0	1
Ier5l	30.33	Immediate early response 5-like	Biological process	0	0	1
Glt6d1	25.65	Glycosyltransferase 6 domain containing 1	Carbohydrate metabolic process, biological process	0	0	1
Cercam	29.73	Cerebral endothelial cell adhesion molecule	Cell adhesion	0	0	1
Brd3	27.3	Bromodomain containing 3	Biological process	0	0	1
Wdr5	27.37	WD repeat domain 5	Regulation of transcription, chromatin modification,	0	0	1

			skeletal system development			
1700101E01Rik	28.91	RIKEN cDNA 1700101E01 gene	Biological process	0	0	1
1700007K13Rik	28.32	RIKEN cDNA 1700007K13 gene	Biological process	0	0	1
1700001O22Rik	30.65	RIKEN cDNA 1700001O22 gene	Biological process	0	0	1
Nrarp	25.04	Notch-regulated ankyrin repeat protein	Multicellular organismal development	0	1	1
Rabepk	34.63	Rab9 effector protein with kelch motifs		0	0	1
Wdr34	29.89	WD repeat domain 34		0	0	1
Surf2	26.77	Surfeit gene 2		0	0	1
Ssna1	25.13	Sjogren's syndrome nuclear autoantigen 1		0	0	1
Nsmf	24.91	NMDA receptor synaptonuclear signaling and neuronal migration factor		0	0	1
Surf1	26.77	Surfeit gene 1		0	0	1
Golga2	32.14	Golgi autoantigen, golgin subfamily a, 2		0	0	1
Swi5	32.13	SWI5 recombination repair homolog (yeast)	Biological process	0	0	1
Ak8	28.56	Adenylate kinase 8	Nucleobase, nucleoside, nucleotide and nucleic acid metabolic process	0	0	1
Ccdc183	25.43	Coiled-coil domain containing 183	Biological process	0	0	1
1700019L03Rik	32.63	RIKEN cDNA 1700019L03 gene	Biological process	0	0	1
Fam129b	32.73	Family with sequence similarity 129, member B	Biological process	0	1	1
Prrc2b	32.01	Proline-rich coiled-coil 2B	Biological process	3	0	1
Tmem8c	26.92	Transmembrane protein 8C	Biological process	2	0	1
Nelfb	25.06	Negative elongation	Transcription,	6	0	1

		factor complex member B	regulation of transcription, negative regulation of transcription			
Fam69b	26.48	Family with sequence similarity 69, member B	Biological process	2	0	1
Cacfd1	26.87	Calcium channel flower domain containing 1	Biological process	1	0	1
Cel	28.41	Carboxyl ester lipase	Lipid catabolic process, ceramide catabolic process	0	0	0
Cutal	34.73	CutA divalent cation tolerance homolog-like	Biological process	0	0	0
Ptrh1	32.63	Peptidyl-tRNA hydrolase 1 homolog (S. cerevisiae)	Translation, biological process	0	0	0
Cstad	30.45	CSA-conditional, T cell activation-dependent protein	Mitochondrial membrane organization	0	0	0
Lcn8	25.51	Lipocalin 8	Transport, response to hormone stimulus, response to protein stimulus	0	0	0
Tor4a	25.05	Torsin family 4, member A	Chaperone mediated protein folding requiring cofactor, biological process	0	0	0
Rnf224	25.09	Ring finger protein 224	Biological process	0	0	0
4933433C11Rik	25.07	RIKEN cDNA 4933433C11 gene	Biological process	0	0	0
Ppp1r26	28.3	Protein phosphatase 1, regulatory subunit 26	Biological process	0	0	0
Prdm12	30.83	PR domain containing 12	Neurogenesis	0	0	0
Tubb4b	25.08	Tubulin, beta 4B class IVB	Microtubule-based process, protein	0	0	0

			polymerization, biological process			
Fam102a	32.39	Family with sequence similarity 102, member A	Biological process	0	0	0
Naif1	32.31	Nuclear apoptosis inducing factor 1	Apoptosis, induction of apoptosis	0	0	0
Fam78a	31.92	Family with sequence similarity 78, member A	Biological process	0	0	0
Aif1l	31.81	Allograft inflammatory factor 1-like	Biological process	0	0	0
Fam73b	30.22	Family with sequence similarity 73, member B	Biological process	0	0	0
Fam163b	26.97	Family with sequence similarity 163, member B	Biological process	0	0	0
Fam166a	25.07	Family with sequence similarity 166, member A	Biological process	0	0	0
Tmem210	25.14	Transmembrane protein 210	Biological process	0	0	0
Tmem203	25.11	Transmembrane protein 203	Biological process	0	0	0
Dnlz	26.2	DNL-type zinc finger	Biological process	0	0	0
Nron	33.64	Non-protein coding RNA, repressor of NFAT		0	0	0
