# Supplementary material for: Genetic Architecture of Group A Streptococcal Necrotizing Soft Tissue Infections in the Mouse
Source: PLoS Pathog. 2016 Jul 11;12(7):e1005732. doi: 10.1371/journal.ppat.1005732 (PMC4939974; doi:10.1371/journal.ppat.1005732)
Supplement: S2 Table — (PDF) [file ppat.1005732.s002.pdf]

S2 Table. Host candidate genes in the mapped QTL for percent weight change, PC1 (GN trait ID: 17527) on mouse Chr 7 between 125 and 131Mb

Gene symbol	Chr 7 (Mb)	Gene description	GO biological process	nsSNPs (B6 vs. D2)	Indels in BXD	Score (0-4)
Acsm3	126.9	Acyl-CoA synthetase medium-chain family member 3	Fatty acid metabolic process, lipid metabolic process, fatty acid biosynthetic process	66	3	4
Tmem159	127.25	Transmembrane protein 159	Biological process	22	2	4
Gprc5b	126.12	G protein-coupled receptor, family C, group 5, member B	G-protein coupled receptor protein signaling pathway, signal transduction, biological process	63	12	4
9030624J02Rik	125.88	RIKEN cDNA 9030624J02 gene	Biological process	146	32	4
Gde1	125.83	Glycerophosphodiester phosphodiesterase 1	Glycerol metabolic process, G-protein coupled receptor protein signaling pathway	7	8	4
Tmc7	125.68	Transmembrane channel-like gene family 7	Biological process	176	23	4
Tmc5	125.74	Transmembrane channel-like gene family 5	Biological process	87	27	4
Knop1	125.99	Lysine rich nucleolar protein 1		105	9	4
Acsm4	126.83	Acyl-CoA synthetase medium-chain family member 4	Fatty acid metabolic process, lipid metabolic process, acyl-CoA metabolic	3	1	3

			process			
Acsm1	126.76	Acyl-CoA synthetase medium-chain family member 1	Fatty acid metabolic process, lipid metabolic process, fatty acid biosynthetic process	19	5	3
Coq7	125.67	Demethyl-Q 7	Protein metabolic process, oxidation reduction, cellular response to oxidative stress	89	3	3
Abca14	127.35	ATP-binding cassette, sub-family A (ABC1), member 14	Biological process	46	9	3
Crym	127.33	Crystallin, mu	Metabolic process, thyroid hormone metabolic process	12	1	3
Zp2	127.28	Zona pellucida glycoprotein 2	Binding of sperm to zona pellucida, single fertilization	25	0	3
2610020H08Rik	126.94	RIKEN cDNA 2610020H08 gene	Biological process	7	2	3
Thumpd1	126.86	THUMP domain containing 1	Biological process	2	1	3
Umod	126.61	Uromodulin	Excretion, chemical homeostasis	10	4	3
Ccp110	125.86	Centriolar coiled coil protein 110	Biological process	81	0	3
Syt17	125.53	Synaptotagmin XVII	Transport, biological process	20	28	3
Arl6ip1	125.26	ADP-ribosylation factor-like 6 interacting protein 1	Cotranslational protein targeting to membrane	18	6	3
Iqck	126	IQ motif containing K	Biological	67	34	3

			process			
Gpr139	126.29	G protein-coupled receptor 139	Activation of phospholipase C activity by G-protein coupled receptor protein signaling pathway coupled to IP3 second messenger	7	14	3
Dnah3	127.07	Dynein, axonemal, heavy chain 3	Microtubule-based movement, biological process	5	1	3
Plk1	129.3	Polo-like kinase 1 (Drosophila)	Cell division, mitosis, cell cycle, protein amino acid phosphorylation	1	0	2
Arhgap17	130.42	Rho GTPase activating protein 17	Calcium ion-dependent exocytosis, actin filament organization, signal transduction	2	0	2
Anks4b	127.32	Ankyrin repeat and sterile alpha motif domain containing 4B	Biological process	3	1	2
Dcun1d3	127	DCN1, defective in cullin neddylation 1, domain containing 3 (S. cerevisiae)	Biological process	0	2	2
Gp2	126.59	Glycoprotein 2 (zymogen granule membrane)	Biological process	21	6	2
Vwa3a	127.88	Von Willebrand factor A domain containing 3A	Biological process	0	3	2
Usp31	128.79	Ubiquitin specific peptidase 31	Biological process	1	0	2
Tnrc6a	130.27	Trinucleotide repeat containing 6a	Regulation of translation, gene silencing	1	0	2

			by RNA, cellular response to starvation			
Rbbp6	130.11	Retinoblastoma binding protein 6	Protein ubiquitination, biological process	1	0	2
Cog7	129.07	Component of oligomeric golgi complex 7	Biological process, protein transport, transport	1	0	2
Cdr2	128.1	Cerebellar degeneration-related 2	Biological process	1	0	2
Aqp8	130.61	Aquaporin 8	Transport, water transport, canalicular bile acid transport	1	0	2
Abca16	127.57	ATP-binding cassette, sub-family A (ABC1), member 16		1	0	2
Eri2	126.93	Exoribonuclease 2	Biological process	33	2	2
Smg1	125.27	SMG1 homolog, phosphatidylinositol 3-kinase-related kinase (C. elegans)	Nuclear-transcribed mRNA catabolic process, nonsense-mediated decay, DNA repair	51	15	2
Pdilt	126.63	Protein disulfide isomerase-like, testis expressed	Spermatogenesis, cell differentiation, multicellular organismal development, cell redox homeostasis	15	4	2
Itpripl2	125.63	Inositol 1,4,5-triphosphate receptor interacting protein-like 2		73	2	2
Dctn5	129.28	Dynactin 5	Biological process	0	0	1
Ndufab1	129.23	NADH dehydrogenase	Electron	0	0	1

		(ubiquinone) 1, alpha/beta subcomplex, 1	transport chain, fatty acid biosynthetic process, lipid biosynthetic process			
Ears2	129.18	Glutamyl-tRNA synthetase 2 (mitochondrial)(putative)	Translation, glutamyl-tRNA aminoacylation	0	0	1
Gga2	129.13	Golgi associated, gamma adaptin ear containing, ARF binding protein 2	Protein transport, vesicle-mediated transport	0	0	1
Scnn1b	129.01	Sodium channel, nonvoltage-gated 1 beta	Regulation of sodium ion transport, wound healing, spreading of epidermal cells	0	0	1
Scnn1g	128.88	Sodium channel, nonvoltage-gated 1 gamma	Regulation of sodium ion transport, wound healing, spreading of epidermal cells	0	0	1
Uqcrc2	127.78	Ubiquinol cytochrome c reductase core protein 2	Transport, electron transport chain, proteolysis, biological process	0	0	1
Acsm2	126.71	Acyl-CoA synthetase medium-chain family member 2	Fatty acid metabolic process, biological process, lipid metabolic process	0	0	1
Acsm5	126.67	Acyl-CoA synthetase medium-chain family member 5	Fatty acid metabolic process, biological process, lipid metabolic	0	0	1

			process			
Hs3st2	128.54	Heparan sulfate (glucosamine) 3-O-sulfotransferase 2	Circadian rhythm	0	0	1
Cacng3	129.82	Calcium channel, voltage-dependent, gamma subunit 3	Transport, calcium ion transport, ion transport	0	0	1
Chp2	129.36	Calcineurin-like EF hand protein 2	Regulation of pH, sodium ion transport	0	0	1
Lcmt1	130.52	Leucine carboxyl methyltransferase 1	C-terminal protein amino acid methylation	0	0	1
Slc5a11	130.36	Solute carrier family 5 (sodium/glucose cotransporter), member 11	Apoptosis, sodium ion transport, carbohydrate transport	0	0	1
Ern2	129.31	Endoplasmic reticulum (ER) to nucleus signalling 2	Apoptosis, transcription, cell cycle arrest, response to stress	0	0	1
Ubfd1	129.21	Ubiquitin family domain containing 1	Biological process	0	0	1
Otoa	128.23	Otoancorin	Sensory perception of sound, biological process	0	0	1
Mettl9	128.18	Methyltransferase like 9	Biological process	0	0	1
Polr3e	128.06	Polymerase (RNA) III (DNA directed) polypeptide E	Response to virus, innate immune response, transcription	0	0	1
Eef2k	127.99	Eukaryotic elongation factor-2 kinase	Protein amino acid phosphorylation	0	0	1
Abca15	127.47	ATP-binding cassette, sub-family A (ABC1), member 15	Biological process	0	0	1
Lyrm1	127.04	LYR motif containing	Biological	0	0	1

		1	process			
Sept1	127.01	Septin 1	Cell cycle, cell division	0	0	1
Palb2	129.25	Partner and localizer of BRCA2	Biological process	0	0	1
Nsmce4a	130.32	Non-SMC element 4 homolog A (S. cerevisiae)	Biological process	0	0	1
Igsf6	128.21	Immunoglobulin superfamily, member 6	Biological process	0	0	1
BC030336	127.88	cDNA sequence BC030336	Biological process, oxidation reduction	0	0	1
Taok2	126.66	TAO kinase 2	Protein amino acid phosphorylation, biological process	0	0	1
Prkcb	129.43	Protein kinase C, beta	Cellular calcium ion homeostasis, regulation of dopamine secretion, regulation of growth	0	1	1
Pdzd9	127.8	PDZ domain containing 9	Biological process	0	1	1
Zkscan2	130.62	Zinc finger with KRAB and SCAN domains 2	Biological process	0	0	0
Slx1b	126.48	SLX1 structure-specific endonuclease subunit homolog B (S. cerevisiae)		0	0	0
Fam57b	126.62	Family with sequence similarity 57, member B	Biological process	0	0	0
Srcap	127.35	Snf2-related CREBBP activator protein		0	0	0
