# Supplementary material for: Genetic Architecture of Group A Streptococcal Necrotizing Soft Tissue Infections in the Mouse
Source: PLoS Pathog. 2016 Jul 11;12(7):e1005732. doi: 10.1371/journal.ppat.1005732 (PMC4939974; doi:10.1371/journal.ppat.1005732)
Supplement: S3 Table — (PDF) [file ppat.1005732.s003.pdf]

S3 Table. Host candidate genes in the mapped QTL for lesion size (GN trait ID: 17525) on mouse Chr 6 between 131.6 and 141.8Mb

Gene symbol	Chr 6 (Mb)	Gene description	GO biological process	nsSNPs (B6 vs. D2)	Indels in BXD	Score (0-4)
Etv6	133.99	Ets variant gene 6 (TEL oncogene)	Regulation of transcription, cell differentiation	103	40	4
Wbp11	136.76	WW domain binding protein 11	mRNA processing, rRNA processing, RNA processing, RNA splicing	90	14	4
Lrp6	134.4	Low density lipoprotein receptor-related protein 6	Endocytosis, multicellular organismal development, Wnt receptor signaling pathway	69	61	4
Arhgdib	136.87	Rho, GDP dissociation inhibitor (GDI) beta		27	8	4
Eps8	137.43	Epidermal growth factor receptor pathway substrate 8	Adult locomotory behavior, actin cytoskeleton reorganization	96	56	4
Atf7ip	136.47	Activating transcription factor 7 interacting protein	Negative regulation of transcription from RNA polymerase II promoter	57	22	4
Emp1	135.31	Epithelial membrane protein 1	Cell growth	48	6	4
Ddx47	134.96	DEAD (Asp-Glu-Ala-Asp) box polypeptide 47	Biological process	68	3	4
Gpr19	134.82	G protein-coupled receptor 19	G-protein coupled receptor protein signaling pathway, signal	11	9	4

			transduction			
Loh12cr1	134.59	Loss of heterozygosity, 12, chromosomal region 1 homolog (human)	Biological process	27	20	4
Gsg1	135.19	Germ cell-specific gene 1	Biological process	24	6	4
Apold1	134.93	Apolipoprotein L domain containing 1		78	4	4
Pik3c2g	139.54	Phosphatidylinositol 3-kinase, C2 domain containing, gamma polypeptide	Cell communication, phosphoinositide-mediated signaling	77	84	3
Gucy2c	136.65	Guanylate cyclase 2c	Response to toxin, intracellular signaling cascade, regulation of cell proliferation	72	32	3
Cdkn1b	134.87	Cyclin-dependent kinase inhibitor 1B	Negative regulation of cellular component movement, negative regulation of epithelial cell proliferation	41	0	3
Grin2b	135.68	Glutamate receptor, ionotropic, NMDA2B (epsilon 2)	Regulation of action potential, synaptic transmission, calcium ion transport	94	133	3
Dusp16	134.67	Dual specificity phosphatase 16	Inactivation of MAPK activity	108	22	3
Rerg	137	RAS-like, estrogen-regulated, growth-inhibitor	Signal transduction, small GTPase mediated signal transduction	70	27	3
Erp27	136.86	Endoplasmic reticulum protein 27	Biological process	90	14	3

Mgp	136.82	Matrix Gla protein	Ossification, cell differentiation, multicellular organismal development	12	0	3
Art4	136.8	ADP-ribosyltransferase 4	Biological process	69	5	3
Hebp1	135.09	Heme binding protein 1	Heme metabolic process	26	12	3
Gprc5d	135.06	G protein-coupled receptor, family C, group 5, member D	G-protein coupled receptor protein signaling pathway, hair cycle, keratinization	27	7	3
Gprc5a	135.02	G protein-coupled receptor, family C, group 5, member A	G-protein coupled receptor protein signaling pathway	61	9	3
Plekha5	140.37	Pleckstrin homology domain containing, family A member 5	Biological process	1	0	3
Ptpro	137.2	Protein tyrosine phosphatase, receptor type, O		91	66	3
Plcz1	139.94	Phospholipase C, zeta 1	Lipid catabolic process, multicellular organismal development, signal transduction	35	14	2
Mgst1	138.09	Microsomal glutathione S-transferase 1	Glutathione metabolic process, response to lipopolysaccharide, response to drug	1	0	2
Pde6h	136.9	Phosphodiesterase 6H, cGMP-specific, cone, gamma	Activation of MAPK activity, positive regulation of G-	17	4	2

			protein coupled receptor protein signaling pathway			
H2afj	136.76	H2A histone family, member J	Nucleosome assembly, biological process	50	0	2
Hist4h4	136.75	Histone cluster 4, H4	Biological process, nucleosome assembly	5	0	2
Aebp2	140.57	AE binding protein 2	Regulation of transcription, chromatin modification	2	0	2
Lmo3	138.31	LIM domain only 3	Regulation of transcription	72	29	2
Mansc1	134.56	MANSC domain containing 1	Biological process	38	13	2
Bcl2l14	134.35	Bcl2-like 14 (apoptosis facilitator)	Regulation of apoptosis	88	13	2
Prb1	132.16	Proline-rich protein BstNI subfamily 1	Biological process	4	0	2
Prp2	132.55	Proline rich protein 2		11	0	2
Smim10l 1	133.07	Small integral membrane protein 10 like 1		0	0	2
8430419 L09Rik	135.15	RIKEN cDNA 8430419L09 gene	Biological process	67	15	2
Crebl2	134.78	cAMP responsive element binding protein-like 2	Regulation of transcription	34	2	2
BC04971 5	136.78	cDNA sequence BC049715	Biological process	91	8	2
Smco3	136.78	Single-pass membrane protein with coiled-coil domains 3	Biological process	63	6	2
Plbd1	136.56	Phospholipase B domain containing 1	Lipid catabolic process	69	24	2
Pde3a	141.2	Phosphodiesterase 3A, cGMP inhibited	Regulation of meiosis, negative regulation of apoptosis	0	0	1
Dera	137.7	2-deoxyribose-5-	Deoxyribonucle	0	0	1

		phosphate aldolase homolog (C. elegans)	otide catabolic process, metabolic process			
Capza3	139.99	Capping protein (actin filament) muscle Z-line, alpha 3	Spermatid development, actin cytoskeleton organization	24	0	1
Igbp1b	138.61	Immunoglobulin (CD79A) binding protein 1b	B cell activation, regulation of signal transduction	11	0	1
Strap	137.68	Serine/threonine kinase receptor associated protein	RNA splicing, mRNA processing, negative regulation of transforming growth factor beta receptor signaling pathway	0	0	1
Pbp2	135.26	Phosphatidylethanolamine binding protein 2	Biological process	17	0	1
Prpmp5	132.26	Proline-rich protein MP5	Biological process	9	0	1
Kap	133.8	Kidney androgen regulated protein		4	0	1
Slc15a5	137.93	Solute carrier family 15, member 5	Peptide transport, protein transport	0	1	1
Slco1a4	141.75	Solute carrier organic anion transporter family, member 1a4	Organic anion transport	0	0	0
Slco1b2	141.58	Solute carrier organic anion transporter family, member 1b2	Ion transport, oligopeptide transport, bile acid and bile salt transport	0	0	0
Slco1c1	141.47	Solute carrier organic anion transporter family, member 1c1	Ion transport, bile acid and salt transport	0	0	0
