# Supplementary material for: Genetic Architecture of Group A Streptococcal Necrotizing Soft Tissue Infections in the Mouse
Source: PLoS Pathog. 2016 Jul 11;12(7):e1005732. doi: 10.1371/journal.ppat.1005732 (PMC4939974; doi:10.1371/journal.ppat.1005732)
Supplement: S4 Table — (PDF) [file ppat.1005732.s004.pdf]

S4 Table. Host candidate genes in the mapped QTL for lesion size (GN trait ID: 17525) on mouse Chr 18 between 49.5 and 56.3Mb

Gene symbol	Chr 18 (Mb)	Gene description	GO biological process	nsSNPs (B6 vs. D2)	Indels in BXD	Score (0-4)
Hsd17b4	50.29	Hydroxysteroid (17-beta) dehydrogenase 4	Very-long-chain fatty acid metabolic process, oxidation reduction	30	2	4
Snx24	53.41	Sorting nexing 24	Protein transport, cell communication	27	10	4
Zfp474	52.78	Zinc finger protein 474	Biological process	10	5	4
Srfbp1	52.63	Serum response factor binding protein 1	Regulation of transcription	30	16	4
Dmxl1	49.99	Dmx-like 1	Biological process	6	2	4
Sncaip	52.93	Synuclein, alpha interacting protein (synphilin)	Regulation of neurotransmitter secretion	3	8	3
Tnfaip8	50.21	Tumor necrosis factor, alpha-induced protein 8	Apoptosis	2	13	3
Ftmt	52.49	Ferritin mitochondrial	Oxidation reduction, cellular iron ion homeostasis	12	0	2
Lox	52.68	Lysyl oxidase	Oxidation reduction, collagen fibril organization, wound healing	4	0	2
Prdm6	53.62	PR domain containing 6	Neurogenesis, negative regulation of transcription, negative regulation of smooth muscle cell differentiation	1	2	2
Zfp608	55.05	Zinc finger protein 608	Biological process	1	0	2
Prr16	51.28	Proline rich 16	Biological	0	4	2

			process			
Dtwd2	49.86	DTW domain containing 2	Biological process	0	1	2
Csnk1g3	54.02	Casein kinase 1, gamma 3	Wnt receptor signaling pathway, protein amino acid phosphorylation	0	0	1
Ppic	53.57	Peptidylprolyl isomerase C	Protein folding	0	0	1
Snx2	53.34	Sorting nexin 2	Protein transport, cell communication	0	0	1
1700034 E13Rik	52.81	RIKEN cDNA 1700034E13 gene	Biological process	0	3	1
Gykl1	52.85	Glycerol kinase-like 1	Spermatogenesis	0	0	0
Hdhd1a	50.73	Haloacid dehalogenase-like hydrolase domain	Metabolic process	0	0	0
Cep120	53.84	Centrosomal protein 120	Interkinetic nuclear migration, cell proliferation	0	0	0
Fam170 a	50.44	Family with sequence similarity 170, member A	Biological process	0	0	0
Thap11	52.62	THAP domain containing 11		0	0	0
