# Supplementary material for: Genetic Architecture of Group A Streptococcal Necrotizing Soft Tissue Infections in the Mouse
Source: PLoS Pathog. 2016 Jul 11;12(7):e1005732. doi: 10.1371/journal.ppat.1005732 (PMC4939974; doi:10.1371/journal.ppat.1005732)
Supplement: S5 Table — (PDF) [file ppat.1005732.s005.pdf]

S5 Table. Relative normalized expression levels of host candidate genes for survival (GN trait ID 17524) on mouse Chr 2, after infection in susceptible BXD strains

Index	Gene symbol	Chr 2 (Mb)	Gene description	Regulation[a]	FDR (< 0.10)
1	Ppapdc3	31.95	Phosphatidic acid phosphatase type 2 domain containing 3	-91.542	0.055
2	Ak1	32.49	Adenylate kinase 1	-46.735	0.058
3	Fam73b	30.22	Family with sequence similarity 73, member B	-33.536	0.072
4	Ccbl1	30.04	Cysteine conjugate-beta lyase 1	-32.234	0.021
5	Rxra	27.53	Retinoid X receptor alpha	-25.478	0.025
6	Gle1	29.79	GLE1 RNA export mediator (yeast)	-24.181	0.009
7	Qsox2	26.06	Quiescin Q6 sulfhydryl oxidase 2	-21.165	0.021
8	Sh3glb2	30.2	SH3-domain GRB2-like endophilin B2	-20.797	0.039
9	Dolpp1	30.25	Dolichyl pyrophosphate phosphatase 1	-19.765	0.016
10	Entpd2	25.25	Ectonucleoside triphosphate diphosphohydrolase 2	-18.072	0.065
11	Exosc2	31.53	Exosome component 2	-18.065	0.009
12	Mvb12b	33.59	Multivesicular body subunit 12B	-17.635	0.078
13	Col5a1	27.74	Collagen, type V, alpha 1	-15.879	0.016
14	Ppp2r4	30.27	Protein phosphatase 2A, regulatory subunit B (PR 53)	-15.865	0.029
15	Nup214	31.83	Nucleoporin 214	-15.382	0.016
16	Prrc2b	32.01	Proline-rich coiled-coil 2B	-15.148	0.034
17	Tsc1	28.5	Tuberous sclerosis 1	-14.441	0.057
18	Fubp3	31.43	Far upstream element (FUSE) binding protein 3	-14.335	0.034
19	Setx	28.98	Senataxin	-14.060	0.039
20	Mapkap1	34.29	Mitogen-activated protein kinase associated protein 1	-12.900	0.047
21	Pomt1	32.09	Protein-O-mannosyltransferase 1	-12.524	0.051
22	Rabepk	34.63	Rab9 effector protein with kelch motifs	-12.185	0.045
23	Rexo4	26.81	REX4, RNA exonuclease 4 homolog (S. cerevisiae)	-11.972	0.016
24	Ciz1	32.22	CDKN1A interacting zinc finger protein 1	-11.599	0.096
25	Surf2	26.77	Surfeit gene 2	-11.588	0.085
26	Rabl6	25.44	RAB, member RAS oncogene family-like 6	-11.205	0.041
27	Ncs1	31.1	Neuronal calcium sensor 1	-10.955	0.058
28	Ptges2	32.25	Prostaglandin E synthase 2	-10.507	0.048
29	Asb6	30.68	Ankyrin repeat and SOCS box-containing protein 6	-10.409	0.029
30	Med27	29.2	Mediator complex subunit 27	-10.372	0.036
31	Sptan1	29.82	Spectrin alpha, non-erythrocytic 1	-9.975	0.033
32	Cacfd1	26.87	Calcium channel flower domain	-9.932	0.045

			containing 1		
33	Zer1	29.95	Zer-1 homolog (C. elegans)	-9.803	0.039
34	Pmpca	26.24	Peptidase (mitochondrial processing) alpha	-9.787	0.060
35	Lmx1b	33.42	LIM homeobox transcription factor 1 beta	-9.752	0.064
36	Tbc1d13	29.99	TBC1 domain family, member 13	-9.686	0.021
37	Gpr107	31.01	G protein-coupled receptor 107	-9.631	0.046
38	Nfatc1	#	Nuclear factor of activated T cells, cytoplasmic, calcineurin dependent 1	-9.376	0.043
39	Swi5	32.13	SWI5 recombination repair homolog (yeast)	-9.123	0.041
40	Anapc2	25.13	Anaphase promoting complex subunit 2	-9.084	0.016
41	Med22	26.76	Mediator complex subunit 22	-8.004	0.009
42	Notch1	26.31	Notch gene homolog 1 (Drosophila)	-7.897	0.030
43	Ehmt1	24.65	Euchromatic histone methyltransferase 1	-7.777	0.025
44	Usp20	30.85	Ubiquitin specific peptidase 20	-7.426	0.033
45	Rapgef1	29.48	Rap guanine nucleotide exchange factor (GEF) 1	-7.340	0.090
46	Dpm2	32.43	Dolichol-phosphate (beta-D) mannosyltransferase 2	-7.236	0.016
47	Slc27a4	29.66	Solute carrier family 27 (fatty acid transporter), member 4	-7.131	0.065
48	Ralgds	28.37	Ral guanine nucleotide dissociation stimulator	-7.110	0.016
49	Tprn	25.12	Taperin	-7.095	0.029
50	Nelfb	25.06	Negative elongation factor complex member B	-7.072	0.025
51	Gapvd1	34.53	GTPase activating protein and VPS9 domains 1	-6.997	0.016
52	Ndor1	25.1	NADPH dependent diflavin oxidoreductase 1	-6.948	0.021
53	Lrsam1	32.78	Leucine rich repeat and sterile alpha motif containing 1	-6.920	0.078
54	Npdc1	25.26	Neural proliferation, differentiation and control gene 1	-6.585	0.043
55	Edf1	25.41	Endothelial differentiation-related factor 1	-5.768	0.049
56	Sdccag3	26.24	Serologically defined colon cancer antigen 3	-5.739	0.049
57	Slc25a25	32.27	Solute carrier family 25 (mitochondrial carrier, phosphate carrier), member 25	-5.737	0.078
58	Tor1b	30.81	Torsin family 1, member B	-5.645	0.023
59	Zbtb43	33.31	Zinc finger and BTB domain	-5.624	0.016

			containing 43		
60	Pnpla7	24.83	Patatin-like phospholipase domain containing 7	-5.587	0.058
61	Dph7	24.82	Diphthamine biosynthesis 7	-5.291	0.087
62	Golga2	32.14	Golgi autoantigen, golgin subfamily a, 2	-5.074	0.023
63	Coq4	29.64	Coenzyme Q4 homolog (yeast)	-4.843	0.078
64	Lrrc8a	30.09	Leucine rich repeat containing 8A	-4.676	0.042
65	Tor2a	32.61	Torsin family 2, member A	-4.088	0.034
66	Abl1	31.54	v-abl Abelson murine leukemia oncogene 1	-4.066	0.078
67	Cercam	29.73	Cerebral endothelial cell adhesion molecule	-4.033	0.053
68	Surf6	26.75	Surfeit gene 6	-3.302	0.092
69	Sh2d3c	32.58	SH2 domain containing 3C	-2.968	0.062
70	Ntng2	29.05	Netrin G2	-2.693	0.091
71	Wdr34	29.89	WD repeat domain 34	-2.137	0.025
72	Lcn9	25.68	Lipocalin 9	2.521	0.065
73	Ass1	31.33	Argininosuccinate synthetase 1	4.198	0.092

[a] Genes with positive values are up regulated, while negative values are down regulated
P values were calculated through t-test, from which FDR were computed through R studio
[#] Nfatc1 was tested instead of Nron (non-protein coding RNA, repressor of NFAT)
