# Supplementary material for: Genetic Architecture of Group A Streptococcal Necrotizing Soft Tissue Infections in the Mouse
Source: PLoS Pathog. 2016 Jul 11;12(7):e1005732. doi: 10.1371/journal.ppat.1005732 (PMC4939974; doi:10.1371/journal.ppat.1005732)
Supplement: S6 Table — (PDF) [file ppat.1005732.s006.pdf]

S6 Table. Relative normalized expression levels of host candidate genes for percent weight change, PC1 (GN trait ID: 17527) on mouse Chr 7, after infection in susceptible BXD strains

Index	Gene symbol	Chr 7 (Mb)	Gene description	Regulation[a]	FDR (< 0.10)
1	Uqcrc2	127.78	Ubiquinol cytochrome c reductase core protein 2	-51.755	0.043
2	Zp2	127.28	Zona pellucida glycoprotein 2	-48.651	0.014
3	Crym	127.33	Crystallin, mu	-43.493	0.039
4	Coq7	125.67	Demethyl-Q 7	-42.094	0.036
5	Eef2k	127.99	Eukaryotic elongation factor-2 kinase	-30.122	0.071
6	Lyrm1	127.04	LYR motif containing 1	-26.497	0.078
7	Gga2	129.13	Golgi associated, gamma adaptin ear containing, ARF binding protein 2	-22.563	0.063
8	Tmem159	127.25	Transmembrane protein 159	-19.842	0.007
9	BC030336	127.88	cDNA sequence BC030336	-19.556	0.039
10	Lcmt1	130.52	Leucine carboxyl methyltransferase 1	-15.918	0.039
11	Srcap	127.35	Snf2-related CREBBP activator protein	-15.066	0.016
12	Rbbp6	130.11	Retinoblastoma binding protein 6	-14.372	0.016
13	Nsmce4a	130.32	Non-SMC element 4 homolog A (S. cerevisiae)	-13.667	0.025
14	Cdr2	128.1	Cerebellar degeneration-related 2	-10.953	0.058
15	Dctn5	129.28	Dynactin 5	-10.277	0.025
16	Tnrc6a	130.27	Trinucleotide repeat containing 6a	-10.027	0.045
17	9030624J02Rik	125.88	RIKEN cDNA 9030624J02 gene	-9.524	0.041
18	Dcun1d3	127	DCN1, defective in cullin neddylation 1, domain containing 3 (S. cerevisiae)	-9.334	0.054
19	Knop1	125.99	Lysine rich nucleolar protein 1	-9.099	0.026
20	Thumpd1	126.86	THUMP domain containing 1	-8.768	0.033
21	Smg1	125.27	SMG1 homolog, phosphatidylinositol 3-kinase-related kinase (C. elegans)	-8.682	0.009
22	Arl6ip1	125.26	ADP-ribosylation factor-like 6 interacting protein 1	-8.302	0.044
23	Hs3st2	128.54	Heparan sulfate (glucosamine) 3-O-sulfotransferase 2	-7.600	0.024
24	Cog7	129.07	Component of oligomeric golgi complex 7	-6.992	0.055
25	Gde1	125.83	Glycerophosphodiester phosphodiesterase 1	-6.475	0.055
26	Ndufab1	129.23	NADH dehydrogenase (ubiquinone) 1, alpha/beta subcomplex, 1	-6.250	0.044
27	Ccp110	125.86	Centriolar coiled coil protein 110	-4.986	0.034
28	Arhgap17	130.42	Rho GTPase activating protein 17	-4.223	0.033
29	Chp2	129.36	Calcineurin-like EF hand protein 2	2.664	0.091

[a] Genes with positive values are up regulated, while negative values are down regulated
P values were calculated through t-test, from which FDR were computed through R studio
