# Supplementary material for: Genetic Architecture of Group A Streptococcal Necrotizing Soft Tissue Infections in the Mouse
Source: PLoS Pathog. 2016 Jul 11;12(7):e1005732. doi: 10.1371/journal.ppat.1005732 (PMC4939974; doi:10.1371/journal.ppat.1005732)
Supplement: S7 Table — (PDF) [file ppat.1005732.s007.pdf]

S7 Table. Relative normalized expression levels of host candidate genes for lesion size (GN trait ID: 17525) on mouse Chr 18, after infection in susceptible BXD strains

Index	Gene symbol	Chr	Mb	Gene description	Regulation[a]	FDR (< 0.10)
1	Eps8	6	137.43	Epidermal growth factor receptor pathway substrate 8	-18.064	0.040
2	Crebl2	6	134.78	cAMP responsive element binding protein-like 2	-16.006	0.018
3	Plekha5	6	140.37	Pleckstrin homology domain containing, family A member 5	-15.677	0.028
4	Lrp6	6	134.4	Low density lipoprotein receptor-related protein 6	-12.575	0.033
5	Atf7ip	6	136.47	Activating transcription factor 7 interacting protein	-11.929	0.009
6	Dera	6	137.7	2-deoxyribose-5-phosphate aldolase homolog (C. elegans)	-10.359	0.020
7	Plbd1	6	136.56	Phospholipase B domain containing 1	-8.841	0.060
8	Aebp2	6	140.57	AE binding protein 2	-8.190	0.030
9	Ptpro	6	137.2	Protein tyrosine phosphatase, receptor type, O	-7.666	0.078
10	Wbp11	6	136.76	WW domain binding protein 11	-6.658	0.025
11	Dusp16	6	134.67	Dual specificity phosphatase 16	-5.782	0.072
12	Etv6	6	133.99	Ets variant gene 6 (TEL oncogene)	-5.492	0.016
13	Smim10l1	6	133.07	Small integral membrane protein 10 like 1	-4.896	0.092
14	Hebp1	6	135.09	Heme binding protein 1	-3.863	0.025
15	Dmxl1	18	49.99	Dmx-like 1	-29.241	0.016
16	Snx24	18	53.41	Sorting nexing 24	-15.252	0.048
17	Ppic	18	53.57	Peptidylprolyl isomerase C	-13.394	0.024
18	Hsd17b4	18	50.29	Hydroxysteroid (17-beta) dehydrogenase 4	-12.085	0.040
19	Srfbp1	18	52.63	Serum response factor binding protein 1	-10.725	0.040
20	Csnk1g3	18	54.02	Casein kinase 1, gamma 3	-9.661	0.011
21	Snx2	18	53.34	Sorting nexin 2	-7.476	0.072
22	Zfp608	18	55.05	Zinc finger protein 608	-4.313	0.034
23	Gykl1	18	52.85	Glycerol kinase-like 1	2.984	0.064

[a] Genes with positive values are up regulated, while negative values are down regulated
P values were calculated through t-test, from which FDR were computed through R studio
