# Supplementary material for: Genetic Architecture of Group A Streptococcal Necrotizing Soft Tissue Infections in the Mouse
Source: PLoS Pathog. 2016 Jul 11;12(7):e1005732. doi: 10.1371/journal.ppat.1005732 (PMC4939974; doi:10.1371/journal.ppat.1005732)
Supplement: S8 Table — (PDF) [file ppat.1005732.s008.pdf]

S8 Table. List of genes in the functional gene network given by IPA

Symbol	Entrez Gene Name
ACAA1	Acetyl-CoA acyltransferase 1
ACSL1	Acyl-CoA synthetase long-chain family member 1
ALDH1A1	Aldehyde dehydrogenase 1 family, member A1
ASS1	Argininosuccinate synthase 1
BRAF	B-Raf proto-oncogene, serine/threonine kinase
CCL17	Chemokine (C-C motif) ligand 17
CCL20	Chemokine (C-C motif) ligand 20
CCNB1	Cyclin B1
CDKN1A	Cyclin-dependent kinase inhibitor 1A (p21, Cip1)
CEBPB	CCAAT/enhancer binding protein (C/EBP), beta
CIZ1	CDKN1A interacting zinc finger protein 1
COL5A1	Collagen, type V, alpha 1
COQ7	Coenzyme Q7 homolog, ubiquinone (yeast)
CREBL2	cAMP responsive element binding protein-like 2
DAXX	Death-domain associated protein
DPM2	Dolichyl-phosphate mannosyltransferase polypeptide 2, regulatory subunit
EHMT1	Euchromatic histone-lysine N-methyltransferase 1
ENTPD2	Ectonucleoside triphosphate diphosphohydrolase 2
EOMES	Eomesodermin
ESR2	Estrogen receptor 2 (ER beta)
ETV6	Ets variant 6
FABP4	Fatty acid binding protein 4, adipocyte
FAS	Fas cell surface death receptor
GLI1	GLI family zinc finger 1
GOLGA2	Golgin A2
GPR84	G protein-coupled receptor 84
HDAC1	Histone deacetylase 1
HDAC7	Histone deacetylase 7
HEBP1	Heme binding protein 1
HOXD10	Homeobox D10
HSD17B4	Hydroxysteroid (17-beta) dehydrogenase 4
IgG	--
IL1B	Interleukin 1, beta
IL4	Interleukin 4
LMX1B	LIM homeobox transcription factor 1, beta
MAD2L1	MAD2 mitotic arrest deficient-like 1 (yeast)
MAP3K8	Mitogen-activated protein kinase kinase kinase 8
MCM3	Minichromosome maintenance complex component 3
MTOR	Mechanistic target of rapamycin (serine/threonine kinase)
MYOD1	Myogenic differentiation 1
NCS1	Neuronal calcium sensor 1
NDUFAB1	NADH dehydrogenase (ubiquinone) 1, alpha/beta subcomplex, 1, 8kDa
NELFB	Negative elongation factor complex member B
NFE2L2	Nuclear factor, erythroid 2-like 2

PAX6	Paired box 6
PCNA	Proliferating cell nuclear antigen
PDGFRB	Platelet-derived growth factor receptor, beta polypeptide
PDK4	Pyruvate dehydrogenase kinase, isozyme 4
PLEKHA5	Pleckstrin homology domain containing, family A member 5
PLSCR1	Phospholipid scramblase 1
PPARD	Peroxisome proliferator-activated receptor delta
PTEN	Phosphatase and tensin homolog
PTPRO	Protein tyrosine phosphatase, receptor type, O
RABL6	RAB, member RAS oncogene family-like 6
RAF1	Raf-1 proto-oncogene, serine/threonine kinase
RAG2	Recombination activating gene 2
RBBP6	Retinoblastoma binding protein 6
RICTOR	RPTOR independent companion of MTOR, complex 2
RPLP0	Ribosomal protein, large, P0
SETX	Senataxin
SGK1	Serum/glucocorticoid regulated kinase 1
SLC25A25	Solute carrier family 25 (mitochondrial carrier; phosphate carrier), member 25
SREBF1	Sterol regulatory element binding transcription factor 1
TICAM1	Toll-like receptor adaptor molecule 1
TNRC6A	Trinucleotide repeat containing 6A
TOB1	Transducer of ERBB2, 1
UQCRC2	Ubiquinol-cytochrome c reductase core protein II
VDR	Vitamin D (1,25- dihydroxyvitamin D3) receptor
ZFP36	ZFP36 ring finger protein
ZNF608	Zinc finger protein 608
